# The papain-like protease determines a virulence trait that varies among members of the SARS-coronavirus species

**DOI:** 10.1371/journal.ppat.1007296

**Published:** 2018-09-24

**Authors:** Daniela Niemeyer, Kirstin Mösbauer, Eva M. Klein, Andrea Sieberg, Robert C. Mettelman, Anna M. Mielech, Ronald Dijkman, Susan C. Baker, Christian Drosten, Marcel A. Müller

**Affiliations:** 1 Institute of Virology, Charité-Universitätsmedizin Berlin, corporate member of Freie Universität Berlin, Humboldt-Universität zu Berlin, and Berlin Institute of Health, Berlin, Germany; 2 Institute of Virology, University of Bonn Medical Centre, Bonn, Germany; 3 German Centre for Infection Research, associated partner Charité, Berlin, Germany; 4 Department of Microbiology and Immunology, Loyola University of Chicago, Maywood, IL, United States of America; 5 Institute of Virology and Immunology, Bern & Mittelhäusern, Switzerland; 6 Department of Infectious Diseases and Pathobiology, University of Bern, Bern, Switzerland; The University of Hong Kong, HONG KONG

## Abstract

SARS-coronavirus (CoV) is a zoonotic agent derived from rhinolophid bats, in which a plethora of SARS-related, conspecific viral lineages exist. Whereas the variability of virulence among reservoir-borne viruses is unknown, it is generally assumed that the emergence of epidemic viruses from animal reservoirs requires human adaptation. To understand the influence of a viral factor in relation to interspecies spillover, we studied the papain-like protease (PLP) of SARS-CoV. This key enzyme drives the early stages of infection as it cleaves the viral polyprotein, deubiquitinates viral and cellular proteins, and antagonizes the interferon (IFN) response. We identified a bat SARS-CoV PLP, which shared 86% amino acid identity with SARS-CoV PLP, and used reverse genetics to insert it into the SARS-CoV genome. The resulting virus replicated like SARS-CoV in Vero cells but was suppressed in IFN competent MA-104 (3.7-fold), Calu-3 (2.6-fold) and human airway epithelial cells (10.3-fold). Using ectopically-expressed PLP variants as well as full SARS-CoV infectious clones chimerized for PLP, we found that a protease-independent, anti-IFN function exists in SARS-CoV, but not in a SARS-related, bat-borne virus. This PLP-mediated anti-IFN difference was seen in primate, human as well as bat cells, thus independent of the host context. The results of this study revealed that coronavirus PLP confers a variable virulence trait among members of the species SARS-CoV, and that a SARS-CoV lineage with virulent PLPs may have pre-existed in the reservoir before onset of the epidemic.

## Author summary

Novel detection and sequencing technologies have greatly improved our knowledge of virus diversity in nature. Metaviromic screening of zoonotic animal reservoirs has become an established approach in pathogen surveillance and pandemic preparedness research. However, knowledge of viral genomes and host-associated viromes is insufficient to predict zoonotic spillover events of reservoir-borne viruses. Phenotypic characterization of important viral functions will be necessary to identify virulence traits that determine the potential of viral emergence. As proof-of-principle, the present study demonstrates relevant functional differences between one of the main host immune antagonists of bat-borne viruses that belong to the same virus species as the epidemic agent of SARS. The antagonist, the papain-like protease, shows double action against IFN-mediated antiviral effects in epidemic SARS-coronavirus (binding and processing of ubiquitin), whereas the homologous protein in bat-borne viruses has only the processing function. This finding is surprising as the papain-like protease is a highly conserved protein domain that was not expected to vary among conspecific coronaviruses. PLP function may represent a variable virulence trait among reservoir-borne viruses. The preformed virulence of the primordial genetic lineage may have supported the emergence of SARS-CoV as a human epidemic agent.

## Introduction

The coronaviruses (CoV, family Coronaviridae) are among the most relevant groups of viruses with zoonotic potential. CoVs are large, positive-sense, single-stranded RNA viruses that cause acute and prolonged infections in a variety of mammals and birds. Pathogenic human CoVs include members of the genus *Alphacoronavirus*, termed human coronavirus (HCoV)-NL63 and HCoV-229E, as well as members of the genus *Betacoronavirus*, termed HCoV-OC43 and HCoV-HKU1. These endemic viruses cause upper and lower respiratory tract infections in humans worldwide. Past zoonotic descent can be inferred for HCoV-OC43 and -229E, respectively [1–4]. Actual zoonotic acquisition is known for two betacoronaviruses that both cause severe lung disease in humans. The Middle East respiratory syndrome coronavirus (MERS-CoV) is a zoonotic agent that is frequently and repeatedly acquired by humans upon contact with dromedary camels in the Arabian Peninsula and parts of Africa [5, 6]. This virus seems to cause only limited human-to-human transmission, but is considered a major threat to global public health due to recurring nosocomial outbreaks that may facilitate onward adaptation to humans [7–9]. The severe acute respiratory syndrome (SARS)-CoV caused an epidemic with sustained human-to-human transmission during 2002 to 2003 in China and other countries, involving more than 8,000 notified infections with a case fatality proportion of about 10% [10, 11].

Human SARS cases were derived from at least two independent zoonotic transmissions from a putative intermediary reservoir in feral carnivores. The majority of cases were part of one continuous chain of human-to-human transmission [12, 13]. Bats are now known to harbor SARS-CoV strains that can directly infect primate cells [14–18]. SARS-CoV has become a paradigmatic subject to study pre-pandemic processes, both on an ecological and molecular level [14, 19–21].

Due to the zoonotic nature of many CoVs, there is an increasing interest to understand functional differences in the relative ways in which viruses deal with host cell defenses across different host species, particularly the innate immune response mediated by type I interferons [22]. The type I interferon (IFN) response is an effective antiviral barrier that may limit zoonotic cross-host infection in general terms [23, 24]. CoV infection is sensed by melanoma differentiation antigen 5 (MDA5) and signaled via mitochondrial antiviral-signaling protein (MAVS), stimulator of IFN genes (STING), and IFN regulatory factor 3 (IRF-3), eventually leading to type I IFN gene transcription [25, 26].

Several IFN antagonist functions of zoonotic SARS-CoV are known. Within the viral structural and accessory proteins, proteins 3b, 6, as well as the nucleocapsid protein have been demonstrated to antagonize type I IFN (e.g., [27]). However, these viral proteins are expressed only after polyprotein processing, nonstructural gene expression, and subgenomic RNA transcription and, therefore, antagonize the downstream effects of IFN receptor signaling rather than the induction of IFN. Distinct from the above SARS-CoV IFN antagonists, the papain-like protease (PLP) is an IFN antagonist that constitutes a domain of the replicase polyprotein and, therefore, may be active at an early stage of the replication cycle to antagonize an upstream step of IFN induction. Additionally, unlike accessory proteins, which can vary greatly between CoV species, maintenance of PLP catalytic activity is critical to viral replication and is therefore conserved across all CoVs [28, 29].

The coronavirus PLP proteins are multifunctional and encode a catalytic triad domain that catalyzes site-specific peptide cleavage of the viral polyprotein and the removal of both ubiquitin and IFN stimulated gene (ISG) 15 post-translational modifications. PLP protease activity catalyzes the processing of the replicase polyprotein at cleavage sites between nsp1/nsp2, nsp2/nsp3, and nsp3/nsp4. PLP deubiquitinating (DUB) activity has been demonstrated in several CoV species, and acts directly and indirectly on several signal molecules in the IRF-3-dependent IFN induction pathway including retinoic acid inducible gene-I (RIG-I), tumor necrosis factor receptor-associated factor 3 (TRAF3), TANK-binding kinase 1 (TBK1) and STING [30, 31]. K63-linked polyubiquitin chains play a general role in signaling cascades of the proinflammatory and IFN systems. K48-linked polyubiquitins label proteins for degradation by the proteasome and activate proinflammatory and antiviral factors. For instance, NFκB is activated by proteasomal degradation of its inhibiting factor IκB [32]. PLP DUB function also involves deISGylating activity, causing the removal of ISG15 modifications from viral and host proteins [33, 34]. ISG15 is an IFN-inducible, antiviral protein that structurally resembles K48-linked di-ubiquitins. It can also be deconjugated by PLPs of other RNA viruses, in particular the ovary tumor (OTU) domain in the papain-like protease 2 (PLP2) of arteriviruses, and the L-gene-encoded OTU domain of nairoviruses [35, 36]. The protease recognition sequence LXGG is common to cleavage sites in the viral protein as well as ubiquitin and its derivatives. The DUB- and deISGylating activities in CoV PLPs should therefore be widely conserved.

Due to the importance of ubiquitin-based innate immune functions, PLP functions may constitute a relevant predictor of the capability of reservoir-borne CoVs to overcome species barriers. PLP activity profiles may differ between relevant zoonotic CoV species. For instance, SARS-CoV has better ability to deconjugate K48-, as opposed to K63-linked polyubiquitins, whereas these activities are balanced in MERS-CoV [37–39]. The processing of ISG15 and K48-linked di-ubiquitin is more effective for SARS- than MERS-CoV PLP [37]. The blocking of induction of IFN by DUB activity was also confirmed for MERS-CoV, but in contrast to SARS-CoV, this inhibition is not independent of PLP’s protease activity [40, 41]. Interestingly, MERS-CoV is more sensitive to the effects of IFN than SARS-CoV [42, 43].

Unfortunately, the PLP activity profile cannot be derived from phylogenetic relatedness. For instance, above-mentioned studies found the distantly related HCoV-NL63 and SARS-CoV to be similar in essential features such as protease-independent, DUB-mediated IFN antagonism, while the MERS-CoV that is much closer related to SARS-CoV only inhibits IFN induction when the protease function is intact (e.g., [34, 37]). Direct studies of PLP functions of reservoir-borne viruses are therefore necessary to help us determine if there are differences in intrinsic virulence or virus-host interactions.

In view of the complexity of PLPs interactions with innate immunity, functional studies have to take the whole viral replication cycle into account. To date, the DUB functions of SARS-CoV PLP have not been studied in the context of a replicating virus. Moreover, no studies have so far compared PLP functions between members of one same viral species including natural variants existing in the zoonotic reservoir. Differences between reservoir-borne and epidemic viruses may uncover mechanisms that aid viral emergence of potentially pandemic strains.

Based on epidemic and reservoir-borne variants of the species SARS-CoV, here we exemplify functional differences in PLP domains. By reverse genetics, we show that the PLP of the epidemic SARS-CoV has an enhanced IFN antagonist function that is independent of PLP protease activity, and that is not present in the PLP of a bat-associated SARS-CoV. Additional mutagenesis studies in replicating virus context associate the functional difference to a more efficient binding of ubiquitin or ubiquitin-like modifiers. Against the general assumption that reservoir-associated viruses are highly adapted to their hosts, we find the PLP of the human epidemic virus to counteract IFN better than the bat-derived PLP even in bat cells. PLP function is a viral virulence trait that varies among reservoir-borne viruses.

## Results

**Fig 1:**
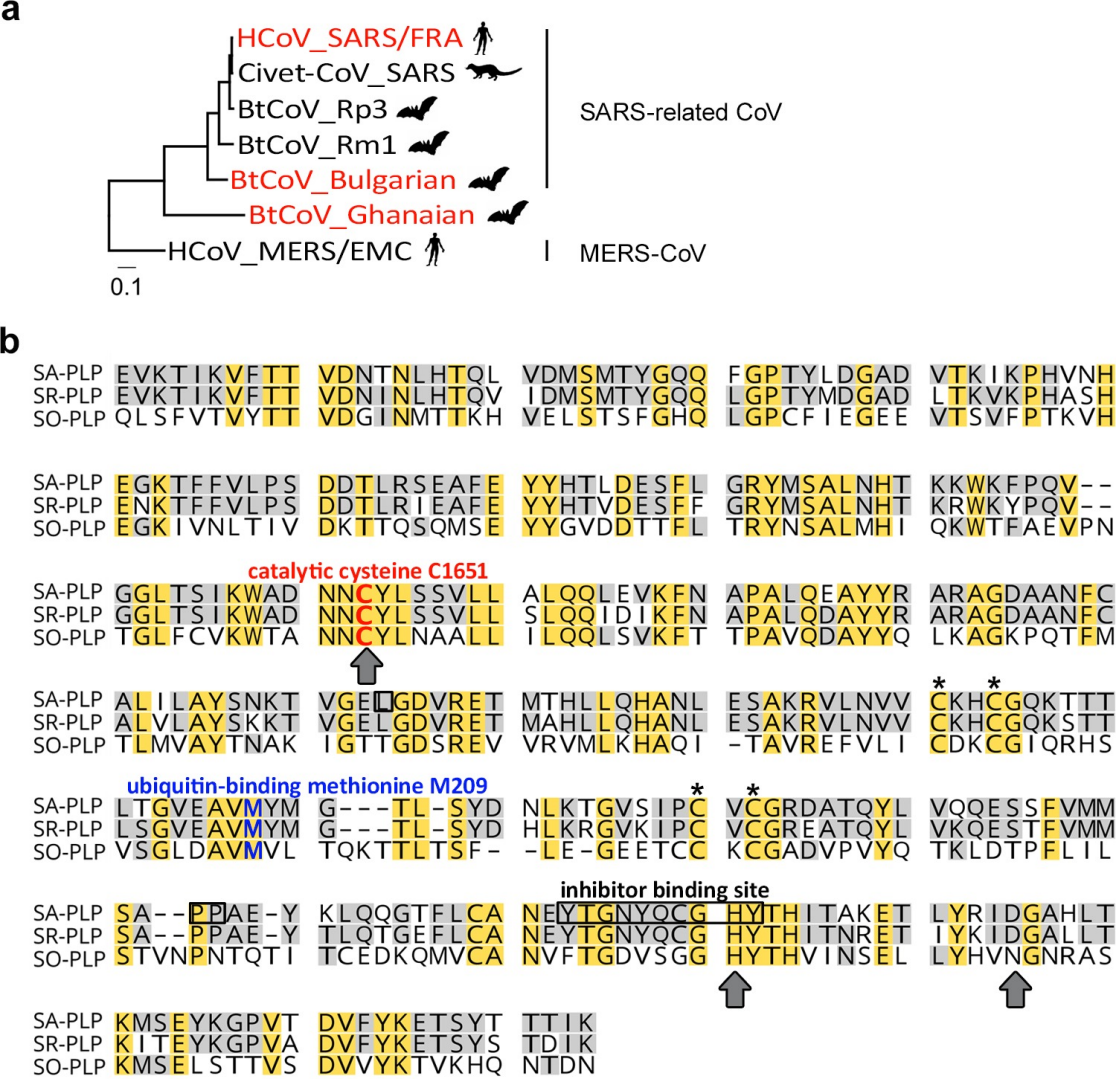
Phylogenetic and sequence-based analysis of the SARS-related bat coronavirus papain-like protease (SR-PLP). (a) Phylogeny of SARS-related beta-CoVs in the *PLP* gene (981 bp fragment) within the nonstructural protein 3. *PLP* genes characterized in the study are colored in red. The right-hand column shows the species classification of the included virus clades according to the International Committee on Taxonomy of Viruses (ICTV). Phylogenetic trees of SARS-related betacoronaviruses (CoVs) were calculated by the Neighbor Joining algorithm in Geneious under the assumption of a Tamura-Nei genetic distance model. Symbols correspond to the respective host species (human, civet and bat). The scale bar refers to the genetic distance. The SARS-outlier CoV (SO-CoV) was identified in a Ghanaian Hipposideros bat. SO-CoV belongs to a novel unclassified beta-CoV species. HCoV: human CoV, FRA: SARS Frankfurt strain, BtCoV: bat CoV. The accession numbers are as follows: HCoV_SARS/FRA: AY310120, Civet CoV_SARS: AY572034, BtCoV_Rp3: DQ071615, BtCoV_Rm1: DQ022305, BtCoV_Bulgarian: GU190215, BtCoV_Ganaian: MG916963, HCoV_MERS/EMC: JX869059. (b) Amino acid sequence alignment for the comparison of SR-PLP to SA-PLP. The alignment is based on the amino acid codes by the Blosum62 algorithm in the Geneious 6 software package. The SO-CoV derived PLP (SO-PLP) was included as an outlier PLP. Yellow boxes indicate conserved residues in all sequences. The boxes in light grey indicate conserved residues in only two sequences. Residues that form the catalytic center are indicated by grey arrows below the sequences. The catalytic cysteine, which was mutated to alanine in the course of this study, is highlighted in red. The ubiquitin-binding methionine at amino acid position 209, which was mutated to arginine (M209R) in this study, is marked in blue. Zinc-binding residues, important for the three dimensional PLP structure, are indicated by asterisks above the sequences. C1651 numeration refers to the position in the SARS-CoV pp1a already used before [46]. Residues framed in black indicate the binding sites of the inhibitor compound 3e, which was used in the course of this study. SA: SARS; SR: Bulgarian; SO-PLP: Ghanaian.

In an earlier study we have described SARS-related CoVs in European (Bulgarian) bat species belonging to the genus *Rhinolophus* [44]. **Fig 1A** shows a phylogeny of SARS-related beta-CoVs based on the *PLP* gene (981 bp fragment, genome position 4885 to 5829 in GenBank accession number AY310120). Based on standing classification criteria, the European bat-derived CoVs are conspecific with human SARS-CoV and in sister relationship to all Asian SARS-related CoVs.

In addition, closely related viruses that were not conspecific with SARS-CoV but represent the closest phylogenetic outgroup to the species SARS-related CoV were discovered in Ghanaian *Hipposideros* bats [45]. *Hipposideros* represents a sister genus to the typical SARS-CoV host *Rhinolophus* (**Fig 1A**). The PLP of human SARS-CoV is henceforth referred to as SA-PLP; the PLP of the conspecific European bat virus as SR-PLP (for SARS-Related); and the PLP of the sister species virus as SO-PLP (for SARS Outgroup).

### Comparison of PLP sequences

An amino acid sequence alignment of the PLP region shows obvious similarities between SA-PLP and SR-PLP, and less so between these PLPs and SO-PLP. The PLP core domains in SA-PLP and SR-PLP each comprise 315 amino acids, and in SO-PLP 320 amino acids. SA-PLP and SR-PLP are 86% (271/315 amino acids) identical. SO-PLP share 39% (125/324 positions including insertions/deletions) and 36% (118/324 positions including insertions/deletions) identical amino acids with SA- and SR-PLP, respectively (**Table 1**). A catalytic triad consisting of the three residues cysteine C1651, histidine H1812 and aspartic acid D1826 was previously shown to be responsible for cleavage of the SARS-CoV replicase polyprotein, and is present in SR-PLP (**Fig 1B**, grey arrows) [46]. In SO-PLP the aspartic acids (D1826) are replaced by an asparagine (N). This alternative type of catalytic domain was previously described for other cysteine proteases [47]. Another indispensable feature of SA-PLP is the zinc-binding domain, comprised of four cysteine residues, which connect the left- and right-hand domains of the papain-like fold by a zinc atom [48]. All these residues are also present within SR- and SO-PLP amino acid sequences **(Fig 1B**, marked with asterisks).

**Table 1:** Amino acid identity and similarity matrix.

PLP	Identity or similarity	% identity or similarity with:		
		SA-PLP	SR-PLP	SO-PLP
**SA-PLP**	**Identity**	**100**		
	**Similarity**	**100**		
**SR-PLP**	**Identity**	**86.03**	**100**	
	**Similarity**	**94.60**	**100**	
**SO-PLP**	**Identity**	**38.58**	**36.42**	**100**
	**Similarity**	**58.64**	**58.95**	**100**

The identities and similarities of the listed proteins is based on an amino acid alignment using the BLOSUM62 substitution matrix and a threshold of 1.

### Comparison of protease activities

**Fig 2:**
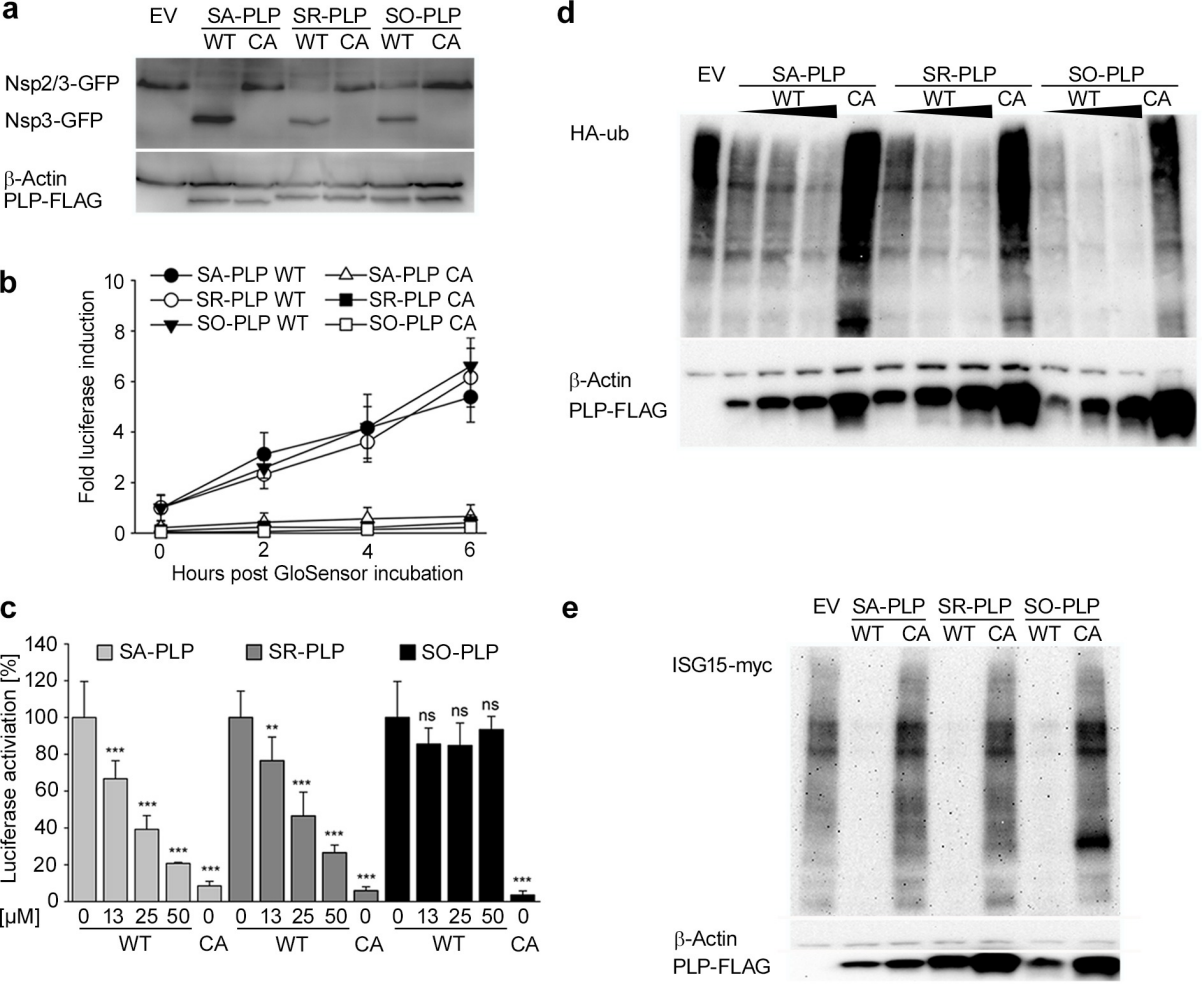
SR-PLP had conserved protease cleavage-, DUB- and deISGylating activities. (a) HEK-293T cells were transfected with empty vector plasmid (EV) or plasmids expressing either wild type (WT)- or catalytic mutant (CA) *PLPs*. SARS-CoV nsp2/3-GFP was cotransfected simultaneously. Lysates were harvested at 16 hours post transfection (hpt), and gene expression was analyzed by Western blotting. (b) A biosensor assay was applied for the detection of PLP cleavage activity. Cells were cotransfected with pGlo *Firefly* luciferase, and either WT-PLP, CA or EV plasmids in 96-wells. At 14 hpi, cells were incubated with GloSensor reagent and luminescence was detected. (c) To investigate the activity spectrum of a SA-PLP protease inhibitor, one hour after the GloSensor incubation, 12.5, 25 or 50 μM of compound 3e or DMSO were added. PLP activities were analyzed in relation to the different amounts of compound 3e at 4 h post treatment. Values were normalized to the respective DMSO-treated WT-PLP. Biosensor assays were performed in triplicate and repeated three times independently. Error bars indicate standard deviations of the means. Statistical significance between DMSO and inhibitor-treated cells or cells transfected with the CA-PLPs, respectively, was determined using one-way ANOVA and Sidak post hoc test. Statistically significant differences are indicated by asterisks (p> 0.05 not significant (ns), p≤ 0.05 significant (*), p≤ 0.01 very significant (**), p≤ 0.001 highly significant (***)). (d) HEK-293T cells were transfected with EV or plasmids expressing either WT- or CA-*PLPs*. For analysis of DUB activity WT-PLP plasmids were transfected in increasing amounts of plasmids (50 ng, 100 ng and 200 ng per 12-well). Control plasmids (200 ng) EV and CA-PLP were transfected for comparison. HA-*ubiquitin* (HA-ub) was coexpressed in all samples. Lysates were harvested at 18 hpt, and gene expression was analyzed by Western blotting. (e) For analysis of deISGylating activity pISG15-myc and the conjugation machinery (UbcH8, Ube1L, and Herc5) of ISG15 were coexpressed in all samples. Lysates were harvested at 18 hpt, and gene expression was analyzed by Western blotting. Western blot experiments were repeated three times independently and one representative blot is shown. β-Actin was applied as loading control.

To functionally compare the PLPs, protease activities were assessed by a *trans*-cleavage assay [49]. The assay was based on coexpression of a SARS-CoV nsp2/3-GFP substrate with the respective PLPs, testing the cleavage of substrate into truncated products nsp2 and nsp3-GFP [49]. During establishment of the assay we noticed a considerable level of mRNA splicing while expressing *SR-PLP* under the control of a chicken β-actin promoter (**S1 Fig**). Codon-optimized constructs were therefore generated. The truncated nsp3-GFP product was detectable by Western blot using anti-GFP epitope tag antibodies with all PLPs (**Fig 2A**; lanes 2, 4 and 6). To confirm that the protease activity of SR-PLP and SO-PLP depended on the same typical catalytic domain as in SA-PLP, the catalytic cysteines of each PLP (C1651) were changed to alanines. For SA-PLP this mutation was previously shown to abolish PLP activity [46]. The mutants are henceforth referred to as CA-mutants. As expected, each PLP CA-mutant was unable to process the SARS nsp2/3-GFP substrate (**Fig 2A**; lanes 3, 5 and 7). To confirm *PLP* expression in all cases, Western blots were done on the same cell lysates using antibodies directed against the FLAG epitope tag fused to PLPs. It was found that the expression levels of all *PLPs* were equal (**Fig 2A,** lower panel).

To enable a quantitative comparison of protease activities, a PLP biosensor luciferase assay was done in which a split *Firefly* luciferase is coexpressed together with the *PLP* of interest. Upon cleavage of an LXGG protease cleavage site in the split luciferase construct, luciferase activity is reconstituted and measured after equilibration with cell membrane-penetrating luciferase substrate [50]. A time-course experiment confirmed that all PLPs had similar protease activities ranging between 1- to 6-fold within 2 to 6 h when compared to CA-mutants corresponding to each PLP (**Fig 2B**).

To investigate if the amino acid differences between the PLPs affect the stereostructure of the catalytic site, a small molecule competitive inhibitor known to be specific for the PLP catalytic site, named 3e [51], was tested side-by-side on the PLPs. Inhibition of protease activity by the inhibitor was successful for both SA-PLP and SR-PLP, indicating structural similarity of both catalytic sites. The inhibitor was slightly more efficient for SA-PLP (EC_50_ = 26.20 μM) than for SR-PLP (EC_50_ = 30.28 μM) which is plausible because the inhibitor was designed to target a beta-loop structure (BL2) of SA-PLP located close to the catalytic site of the protease (**Fig 1B**). The inhibitor had very low efficiency towards SO-PLP, whose inhibitor-binding site has only 46% amino acid identity (6/13 amino acids identical to SA-PLP) to the inhibitor target site in SA-PLP (**Fig 2C**).

### Comparison of DUB functions

To compare DUB activities, HEK-293T cells were cotransfected with increasing doses of plasmids encoding each *PLP* along with constant doses of plasmids encoding HA-tagged ubiquitin. The decrease of protein ubiquitination conferred by PLP was determined by Western blot using anti-HA antibodies. Each PLP deconjugated ubiquitin in a dose-dependent manner suggesting that the PLPs have comparable DUB efficiencies (**Fig 2D**).

Because it is known that SA-PLP has deISGylating activity [34] the efficiency to deconjugate ISG15 from cellular proteins was determined by deISGylation assay. The *PLPs* were coexpressed with myc-tagged ISG15 in HEK-293T cells. The extent of deISGylated proteins was determined by Western blot using anti-myc antibodies. Each PLP was highly efficient in deconjugating ISG15 from the cellular proteins (**Fig 2E**).

Taken together, these results suggested comparable levels of protease activity and identical DUB and deISGylating activities of SA- and SR-PLP when overexpressed in a human cell context. Differential efficiency towards a PLP inhibitor hint at structural differences even between PLPs from conspecific viruses that occur in zoonotic reservoirs. However, complete failure of the inhibitor was only seen with SO-PLP that falls outside the limits of current CoV species classification.

### Protease function but not IFN antagonism is equivalent for SARS-CoV PLPs in full virus context

**Fig 3:**
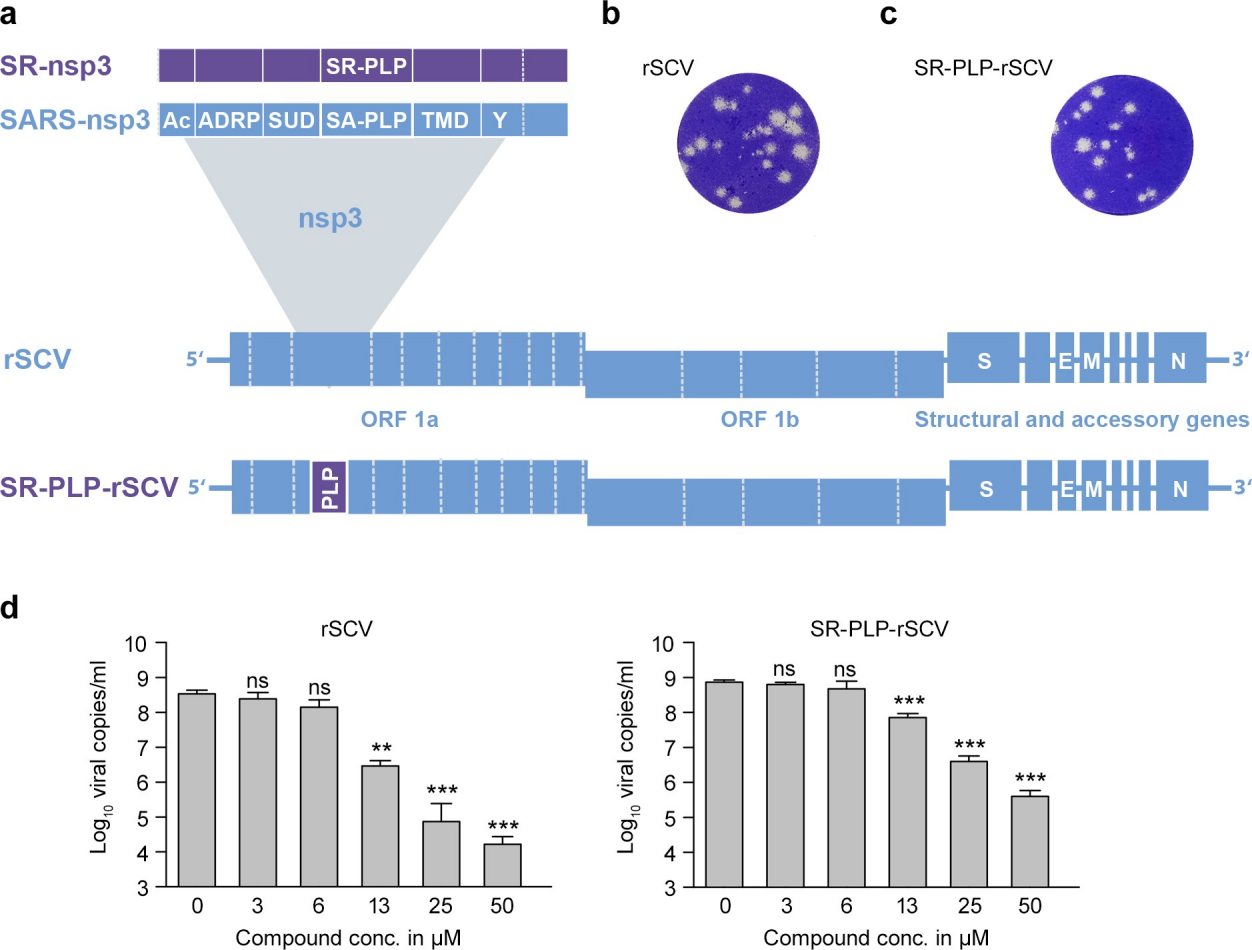
SR-PLP supported viral replication in the context of a recombinant, chimeric SARS-CoV (rSCV). (a) A chimeric rSCV containing *SR-PLP* (SR-PLP-rSCV) was generated by reverse genetics to investigate SR-PLP functions in the context of a replicating virus. *SR-PLP* (purple) was inserted at the genomic position of *SA-PLP* (blue). The plaque morphology was analyzed in Vero E6 cells. Therefore, cells were infected with (b) rSCV and (c) SR-PLP-rSCV (MOI 0.01) and overlaid with a highly viscous medium. At 3 dpi, cells were fixed and stained with crystal violet. (d) To investigate structural integrity of the SR-PLP domain in the molecular context of the SARS-CoV nonstructural protein 3, cells were infected with either rSCV or SR-PLP-rSCV (MOI 0.0001) and treated with the SA-PLP protease inhibitor 3e (24-well format). After 1 h, cells were washed twice with PBS and either DMSO (0 μM) or in DMEM serially diluted compound 3e (3.125, 6.25, 12.5, 25 and 50 μM) was added. Supernatants were collected at 24 hpi. For virus quantification a real-time RT-PCR for genomic SARS-CoV RNA was performed. The experiment was done in triplicate and repeated twice. Error bars indicate the standard deviations of the means. Statistical significance between DMSO- and compound-treated cells was determined using one-way ANOVA and Sidak- or Games-Howell post hoc tests for rSCV and SR-PLP-rSCV, respectively. The 50% effective concentrations (EC_50_) were rSCV: IC_50_ = 2.36 μM and SR-PLP-rSCV: IC_50_ = 11.02 μM.

To quantitatively compare the functions of the two closely related PLPs (SA- and SR-PLP) in the context of the full virus replication cycle, we constructed a chimeric SARS-CoV in which *SA-PLP* was replaced by *SR-PLP* (**Fig 3A**). Viral plaques with similar morphologies were observed for the recombinant wild type virus (rSCV, **Fig 3B**) and the chimeric virus (SR-PLP-rSCV, **Fig 3C**), indicating that the SR-PLP was able to functionally compensate the SA-PLP. As already suggested by protease cleavage assays, SR-PLP-rSCV was slightly less sensitive against protease inhibitor 3e than SA-PLP-rSCV (rSCV: IC_50_ = 2.36 μM; SR-PLP-rSCV: IC_50_ = 11.02 μM), providing additional evidence for functional and structural integrity of SR-PLP in the context of SARS-CoV replication **(Fig 3D)**. To exclude the possibility that 3e was cytotoxic, a cell viability assay was conducted. The number of viable cells decreased with increasing amounts of inhibitor to a minimum of 80% at the highest dose of 50 μM (**S2 Fig**). This confirms the specific action of 3e.

**Fig 4:**
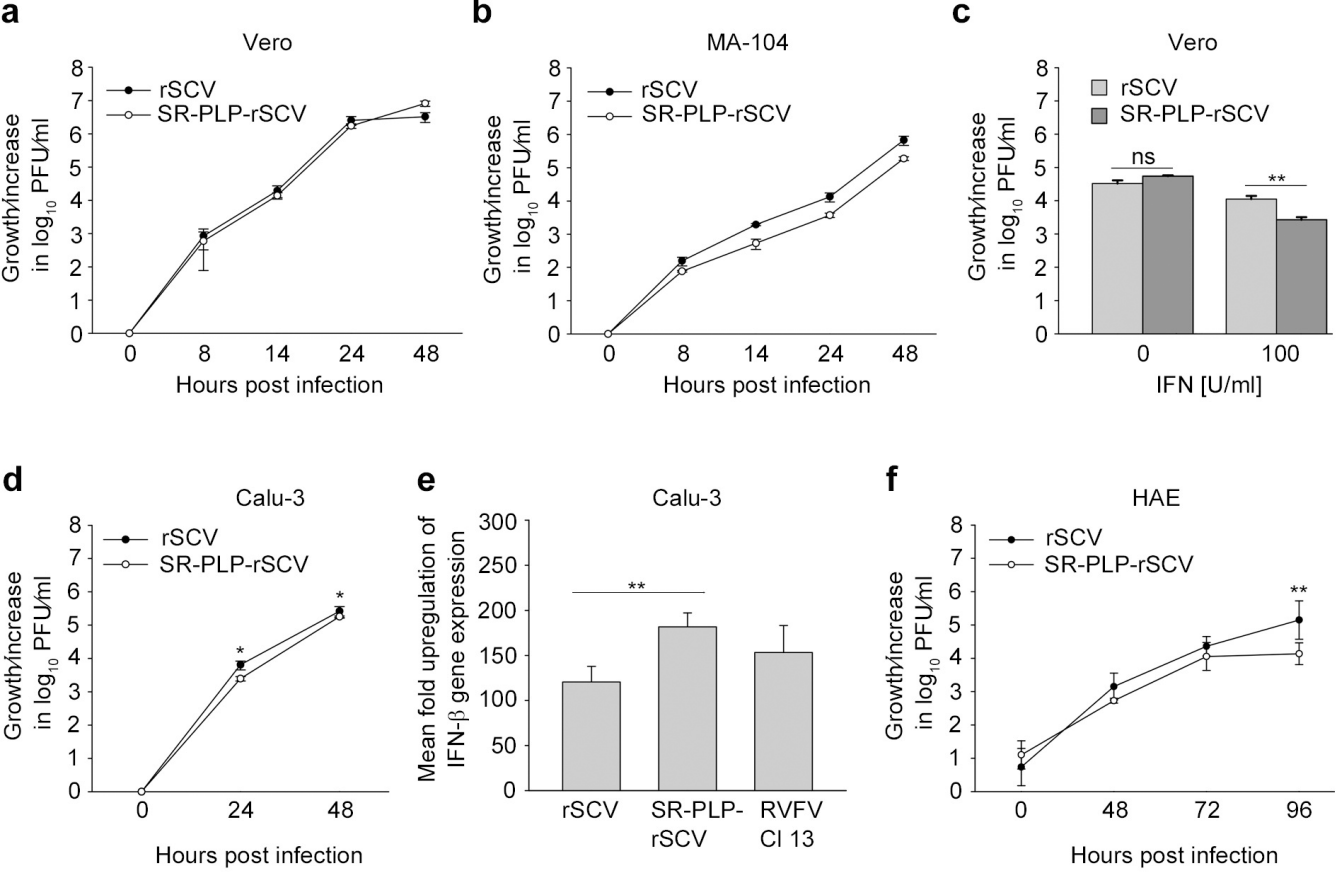
Chimeric SR-PLP-rSCV grew less efficient in presence of type I interferon (IFN). Virus growth was compared in (a) type I IFN-deficient (Vero) primate cells, and (b) IFN-competent (MA-104) primate cells. For virus growth kinetics, cells were infected with rSCV and SR-PLP-rSCV (MOI 0.01). Supernatants were taken at 0, 8, 14, 24 and 48 hpi, and viral replication was determined by a plaque titration assay. Growth experiments in Vero and MA-104 cells were done in triplicate and repeated twice. Error bars indicate the standard deviations of the means. Infectious particle production of rSCV and SR-PLP-rSCV was compared using SPSS Version 23.0.0.0 and a general linear regression model. Growth of both viruses did not significantly differ in Vero cells (p = 0.929 and R-square = 0.670). In MA-104 cells growth of the viruses significantly differed (p = 0.038 and R-square = 0.612). (c) Vero cells were treated with 100 IU/ml of recombinant pan-species IFN-α. At 16 h post IFN treatment, cells were infected with rSCV and SR-PLP-rSCV (MOI 0.01), respectively. Supernatants were taken at 24 hpi, and viral replication was determined by a plaque titration assay. The experiment was performed in triplicate and repeated twice. Error bars indicate the standard deviations of the means. One representative experiment is shown. (d) Virus growth was compared in IFN-competent human cells (Calu-3) as described above. The experiment was performed in triplicate. (e) To determine *IFN-β* expression, Calu-3 cells were infected with rSCV, SR-PLP-rSCV or IFN-inducing RVFV Cl 13 (control of *IFN-β* expression) at an MOI of 1. Total mRNA was extracted from cell lysates at 24 hpi. *IFN-β* expression was determined using quantitative real-time PCR analysis. The mean fold change in *IFN-β* expression was calculated using *TATA-binding protein* (TBP) expression as a reference gene and the 2^−ΔΔCt^ analysis method [55]. The experiment was done in quadruplicates. Statistical significance between the indicated groups was determined using a two-sided t test. (f) Human airway epithelial cells (HAE) were infected with rSCV and SR-PLP-rSCV with an absolute infectious dose of 40,000 PFU. At 0, 48, 72 and 96 hpi samples were taken and viral replication was determined by a plaque titration assay. The experiment was done in duplicate and repeated three times independently. Statistical significance in (d-f) was determined using a two-sided t test.

To obtain a more quantitative comparison of SA- and SR-PLP during virus replication, multistep growth curve experiments were done for both viruses in Vero cells. Both viruses grew to the same titers at all tested time points (range: 8.2x10^2^ PFU/ml to 8.2x10^6^ PFU/ml; 8, 14, 24, 48 hours post infection [hpi], **Fig 4A**). Notably, growth curves differed when both viruses were grown in the type I IFN-competent primate cell line MA-104. SR-PLP-rSCV grew to significantly lower titers than rSCV (general linear regression model, p = 0.038/R-square = 0.612; differences 3.7-fold at 14 hpi and 3.6-fold at 24 hpi, **Fig 4B**). The reduced growth of SR-PLP-rSCV in MA-104 cells may indicate a less efficient viral counteraction against type I IFN.

In order to compare the IFN sensitivity of both viruses, Vero cells were treated with a defined concentration of pan-species IFN-α. A significantly increased IFN sensitivity (4.2-fold at 100 IU/ml; p = 0.008 in a two-sided t test) compared to rSCV confirmed that the anti-IFN activity of SR-PLP was decreased in primate cells **(Fig 4C)**.

To further confirm that growth of SR-PLP-rSCV is also reduced in context of the human cell environment, we performed multistep growth curve experiments in the type I IFN competent human lung epithelial cell line Calu-3 **(Fig 4D)**. Again, SR-PLP-rSCV grew to significantly lower titers than rSCV (2.6 fold at 24 hpi [p = 0.023] and 1.5 fold at 48 hpi [p = 0.016] in a two-sided t test). A 1.5-fold increased detection of *IFN-β* mRNA expression levels in SR-PLP-rSCV- compared to rSCV-infected Calu-3 cells further confirmed that the SR-PLP is less efficient in blocking IFN induction (**Fig 4E**).

To better reflect the human respiratory tract and to generalize the notion that SR-PLP-rSCV grows less efficiently in presence of an active type I IFN response, multistep growth curve experiments were conducted in human airway epithelial cells (HAE). In accordance with our previous studies [42] virus growth was generally delayed in HAE compared to the monoclonal primate and human cell cultures. Importantly, a significantly reduced growth was detected for SR-PLP-rSCV compared to rSCV at 96 hpi (10.3 fold; p = 0.006 in a two-sided t test) providing further confirmation that the anti-IFN activity of SR-PLP may be decreased in type I IFN competent cells **(Fig 4F)**.

### Analysis of differential sensitivity to IFN

**Fig 5:**
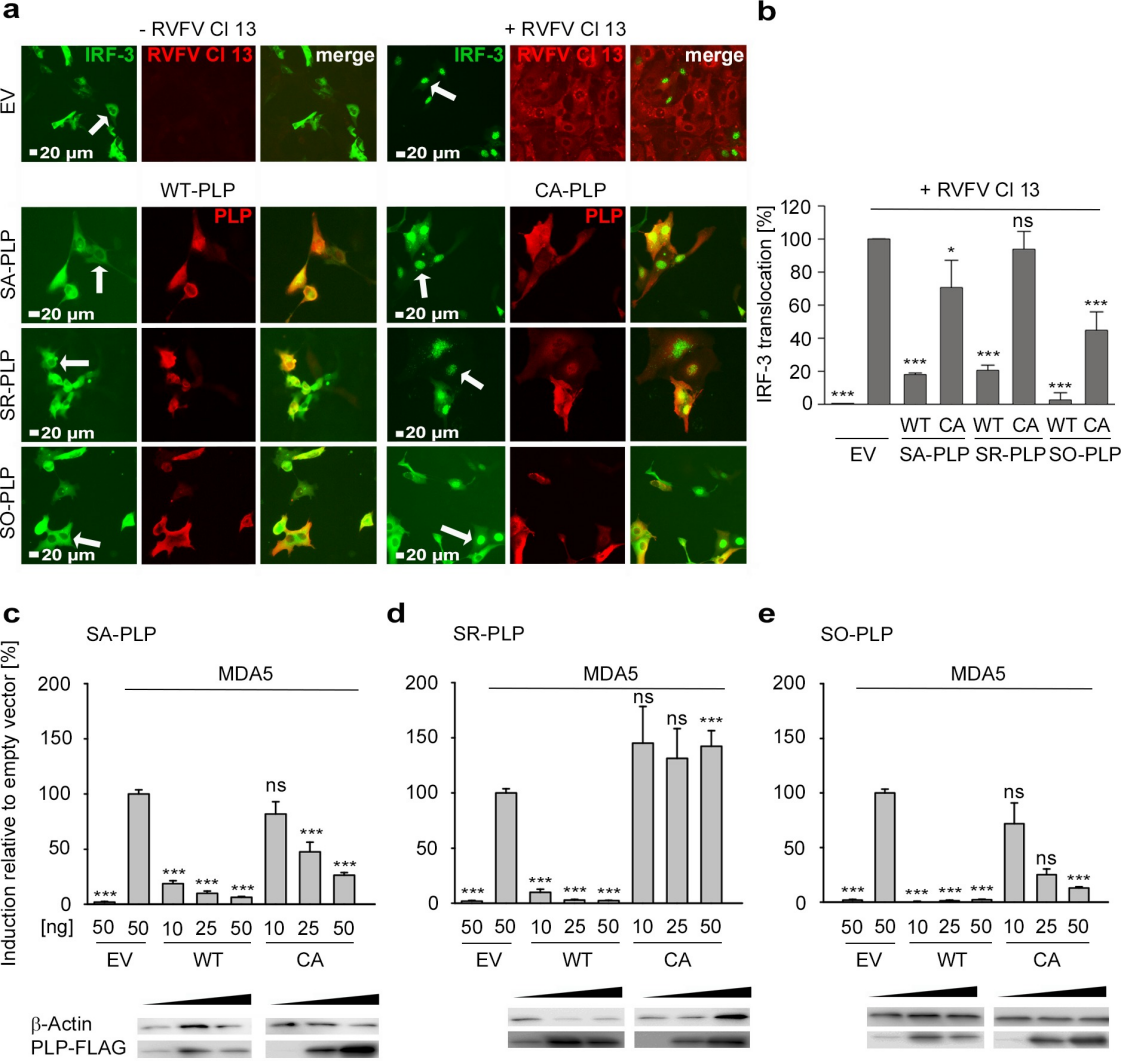
Protease-dependent and protease-independent IFN-antagonistic functions of PLPs. (a) Primate cells (MA-104) were transfected with plasmids expressing 1 μg of WT- or CA-*PLPs*. EV was applied as a control. *GFP-IRF-3* was coexpressed in each sample. At 24 hpi, cells were infected with IFN-inducing, recombinant Rift Valley fever virus clone 13 (RVFV Cl 13) at an MOI of 5. Cells were fixed at 8 hpi. Immunofluorescence pictures show representative results. White arrows indicate representative cells. (b) For quantification of IRF3 translocation events at least 4 representative pictures were taken. The total number of cells positive for GFP-IRF-3 and the respective PLP were determined and the number of GFP-IRF-3 nuclear translocation was calculated. The result represents one of three independently performed experiments. (c, d, e) An IFN-β promoter activation assay was conducted in HEK-293T cells to determine the anti-IFN activities of (c) SA-PLP and (d) SR-PLP and (e) SO-PLP. Cells were transfected with plasmids expressing *MDA5* (IFN stimulator), *Firefly-* (detection of IFN-β promoter activity) and *Renilla-* (detection of the general transcription level) luciferases. 50 ng/24-well of EV were transfected and samples were applied as induction controls. WT- and CA-*PLPs* were coexpressed in a dose-dependent manner (10, 25 and 50 ng/24-well). At 17 hpt, cells were lysed and the luciferase activity was measured. Results are presented as induction relative to EV. The graph shows results of three independently performed experiments, which were all done in triplicate. Expression of the *PLPs* was confirmed by Western blotting. β-Actin was applied as a loading control. The error bars in each graph indicate the standard deviations of the means. Statistical significance between infected/induced EV samples and *PLP*-expressing cells was determined using one-way ANOVA and Dunnett-T3 post hoc test.

The treatment with IFN-α in the above experiment **(Fig 4C)** would broadly affect IFN induction, signaling, and response. Because PLP IFN antagonism functions have been linked to IRF-3 function, a sensitive assay for IRF-3 nuclear translocation was established. Nuclear translocation of an overexpressed IRF-3/GFP fusion protein was stimulated by superinfection with Rift Valley fever virus clone 13 (RVFV Cl 13), an RVFV-mutant devoid of the IFN induction antagonist NSs. RVFV Cl 13 is known to trigger a strong IFN response [52, 53]. The proportion of cells with nuclear translocation of GFP signal was counted microscopically. As summarized in **Fig 5A and 5B**, ectopic expression of *SA-* and *SR-PLP* blocked the nuclear translocation of IRF-3 to comparable levels (SA-PLP: 18% and SR-PLP: 20% of cellular IRF-3/GFP fusion proteins located in the nucleus). The SO-PLP of the outlying virus blocked the nuclear translocation of IRF-3 even more efficiently (3% of cellular IRF-3/GFP fusion proteins located in the nucleus). For all PLPs, CA-mutants were included in the experiment to determine whether anti-IFN effects depended on protease function. The inhibitory capacity of the SA- and SO-PLP CA-mutants were strongly reduced, but still detectable at significant levels (SA-PLP: 71% and SO-PLP: 45% of cells with IRF-3/GFP fusion protein located in the nucleus). In contrast, the SR-PLP CA-mutant had essentially lost its ability to block IRF-3 nuclear translocation (94% of cells with nuclear IRF-3/GFP signal).

To see whether the PLP activity against IRF-3 translocation determined IFN counteraction, the abilities to block IFN promoter activation were compared in a reporter gene assay. All PLPs efficiently blocked IFN-β promoter activation in a dose-dependent manner (6.3%, 2.3% and 2.2% residual IFN-β promoter activation, respectively, at plasmid concentrations of 50 ng for SA-PLP, SR-PLP and SO-PLP; **Fig 5C–5E**). However, differences were observed with CA-mutants. The SA-PLP and SO-PLP CA-mutants still blocked the IFN-β promoter activity in a dose-dependent manner, albeit with reduced efficiency as compared to wild type (SA-PLP: 26.4% [**Fig 5C**] and SO-PLP: 12.9% [**Fig 5E**] residual IFN-β promoter activation at a plasmid concentration of 50 ng). The SR-PLP CA-mutant lost any significant blocking functions against IFN-β promoter activation (**Fig 5D**).

The differential capabilities of SA-PLP, SR-PLP and SO-PLP to counteract IFN induction may be attributable to differential capabilities to inactivate ubiquitin or ubiquitin-like modifiers such as ISG15. SR-PLP may have to rely on protease-dependent cleavage, while SA-PLP and SO-PLP may be able to provide additional IFN-antagonistic activity by binding of ubiquitin or ubiquitin-like modifiers.

### SA-PLP’s protease-independent IFN-antagonistic activity depends on the interaction between SA-PLP and ubiquitin

To understand if within the SARSr-CoV species the SA-PLP as compared to SR-PLP provides additional anti-IFN activity depending on ubiquitin-binding but not cleavage, we decided to introduce a modification in the ubiquitin-binding surface of SA-PLP that should not affect the protease-processing activity. Amino acid residue M209 of SA-PLP interacts directly with a binding patch comprised of I44, V70, L8, R167 and D168 of ubiquitin based on co-crystallization studies [38, 54]. In the MERS-CoV PLP, replacement of methionine at a corresponding position by the bulkier arginine residue was found to prevent the interaction between ectopically expressed PLP and ubiquitin [41]. We thus decided to study the effect of a mutation that selectively destabilizes ubiquitin-binding in SARS-CoV via an M209R mutation.

**Fig 6:**
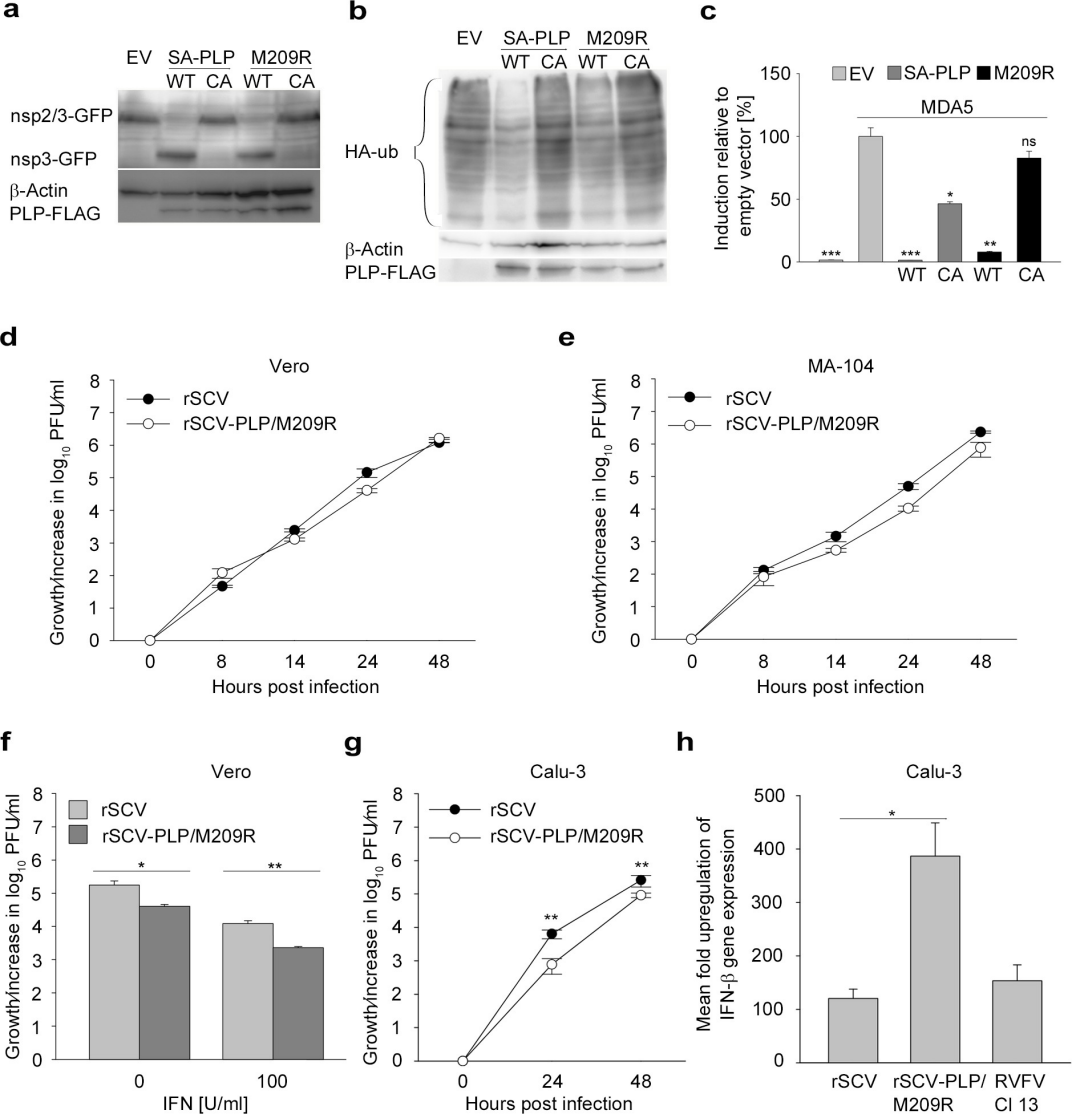
The protease-independent IFN-antagonistic activity of SA-PLP is related to the interaction between SA-PLP and ubiquitin. (a) A *trans*-cleavage assay was conducted in HEK-293T cells to determine the catalytic activity of SA-PLP and the SA-PLP mutant M209R, which had the ubiquitin-binding residue methionine (M) at amino acid position 209 mutated to arginine (R). (b) For analysis of DUB activity, HEK-293T cells were transfected with HA-ub and 200 ng of plasmids expressing either SA-PLP, the SA-PLP mutant M209R or the respective CA-mutants. Lysates were harvested at 18 hpt, and gene expression was analyzed by Western blotting. (c) IFN-β promoter activity was determined using an IFN-β promoter activation assay. 50 ng of SA-PLP encoding plasmids were coexpressed with the luciferase genes and IFN-inducing MDA5. Statistical significance between infected/induced EV samples and *PLP*-expressing cells was determined using one-way ANOVA and Dunnett-T3 post hoc test. Virus growth of rSCV and the modified rSCV-PLP/M209R, carrying the M209R mutation, was compared in type I IFN-deficient primate cells (Vero, d), and IFN-competent primate cells (MA-104, e). For virus growth kinetics, cells were infected with rSCV and rSCV-PLP/M209R at an MOI of 0.01. Each experiment was performed in triplicate. MA-104 growth experiment was repeated twice. Particle production of rSCV and rSCV-PLP/M209R was compared using SPSS Version 23.0.0.0 and a general linear regression model. Growth of both viruses did not significantly differ in Vero cells (p = 0.613 and R-square = 0.020). In MA-104 cells growth of the viruses significantly differed (p = 0.034 and R-square = 0.217). (f) In order to determine the sensitivity of rSCV and rSCV-PLP/M209R towards type I IFN, Vero cells were treated with 100 IU/ml of recombinant pan-species IFN-α. At 16 h post treatment, cells were infected with rSCV or rSCV-PLP/M209R (MOI 0.01) for 24 h. The experiment was performed in triplicate. Statistical significance between the indicated groups was determined using a two-sided t test. (g) Virus growth was compared in human-derived, IFN-competent cells (Calu-3). Cells were infected with rSCV or rSCV-PLP/M209R at an MOI of 0.01. The experiment was done in triplicate. Statistical significance was determined using a two-sided t test. (h) To determine IFN-β expression, Calu-3 cells were infected with rSCV, rSCV-PLP/M209R or IFN-inducing RVFV Cl 13 (control of *IFN-β* expression) at an MOI of 1. Total mRNA was extracted from cell lysates at 24 hpi. *IFN-β* expression was determined using quantitative real-time PCR analysis. The mean fold change in *IFN-β* expression was calculated using *TATA-binding protein* (TBP) expression as a reference gene and the 2^−ΔΔCt^ analysis method [55]. The experiment was done in quadruplicates. Statistical significance between the indicated groups was determined using a two-sided t test.

In order to specifically focus on the consequences of the mutation on IFN antagonism, we had to exclude that M209R disturbs the protease activity of the PLP. Expression plasmids were generated, and protease activities were compared by *trans*-cleavage assays, confirming that the mutation had no impact on the cleavage activity **(Fig 6A)**. M209R PLP processed the nsp2/3 substrate with the same efficiency as SA-PLP.

To confirm that ubiquitin-binding of SA-PLP is reduced by the M209R mutation, a DUB assay was conducted with wild type and M209R SA-PLP expression plasmids as previously described. DUB efficiency of M209R PLP (**Fig 6B**, lane 4) was reduced compared to the wild type PLP (**Fig 6B**, lane 2) as indicated by the increased number of ubiquitinated protein bands in lane 4.

The reduced ubiquitin-binding efficiency should result in a decreased IFN-antagonistic activity of M209R PLP. We thus compared IFN-β promoter activation in a reporter gene assay. Both, the M209R and the corresponding M209R CA mutant, had less anti-IFN activity compared to SA-PLP **(Fig 6C)**. The combination of M209R and CA (**Fig 6C**, right black column) resulted in the complete depletion of significant anti-IFN activity.

To explore the consequences of the disturbed ubiquitin-binding on virus growth, the M209R mutation was introduced into rSCV. As expected, the resulting virus, termed rSCV-PLP/M209R, grew to titers similar to rSCV in a multistep growth curve in Vero cells **(Fig 6D**). As observed with SR-PLP, the congruence with wild type changed when both viruses were grown in type I IFN competent MA-104 cells. In MA-104 cells rSCV-PLP/M209R grew to significantly lower titers than rSCV (general linear regression model, p = 0.034/R-square = 0.217; differences 1.9-fold at 14 hpi and 3.5-fold at 24 hpi, **Fig 6E**).

To confirm that the destabilizing mutation in the ubiquitin-binding domain caused increased IFN sensitivity, Vero cells were pretreated with 100 IU of pan-species IFN-α **(Fig 6F)**. Upon treatment, the growth difference between both viruses was augmented, confirming a significantly increased IFN sensitivity of rSCV-PLP/M209R (4.3-fold at 0 IU/ml [p = 0.023] and 5.2-fold at 100 IU/ml [p = 0.007] in a two-sided t test).

To further confirm the type I IFN-dependent growth differences of rSCV and rSCV-PLP/M209R, multistep virus growth kinetics were done in the human lung epithelial cell line Calu-3 **(Fig 6G)**. Compared to wild type virus-infected cells, the M209R mutation led to significantly lower titers in rSCV-PLP/M209R-infected Calu-3 cells (8.3 fold at 24 hpi [p = 0.007] and 2.8 fold at 48 hpi [p = 0.003] in a two-sided t test).

In order to directly detect the virus-dependent effect on *IFN-β* gene expression, Calu-3 cells were infected with both viruses with an MOI of 1 for 24 h and *IFN-β mRNA* upregulation was detected by quantitative real time RT-PCR assay **(Fig 6H)**. To determine the fold-induction of the *IFN-β* mRNA, the 2^−ΔΔCt^ method was applied with *TATA binding protein* (TBP) as housekeeping gene [55]. The rSCV-PLP/M209R-infected cells had a more pronounced *IFN-β* mRNA expression level (3.2-fold difference between rSCV and rSCV-PLP/M209R at 24 hpi; p = 0.031 in a two-sided t test) compared to rSCV. The type I IFN-inducing RVFV Cl 13 was included as an *IFN-β* mRNA expression control showing that the cells had a fully functional IFN response.

The phenotypic similarity between SR-PLP-rSCV and rSCV-PLP/M209R suggests that SA-PLP, but not SR-PLP, provides additional IFN antagonism via binding of ubiquitin or ubiquitin-like modifiers.

### Differential IFN counteraction in cells of the SARS-CoV natural host

**Fig 7:**
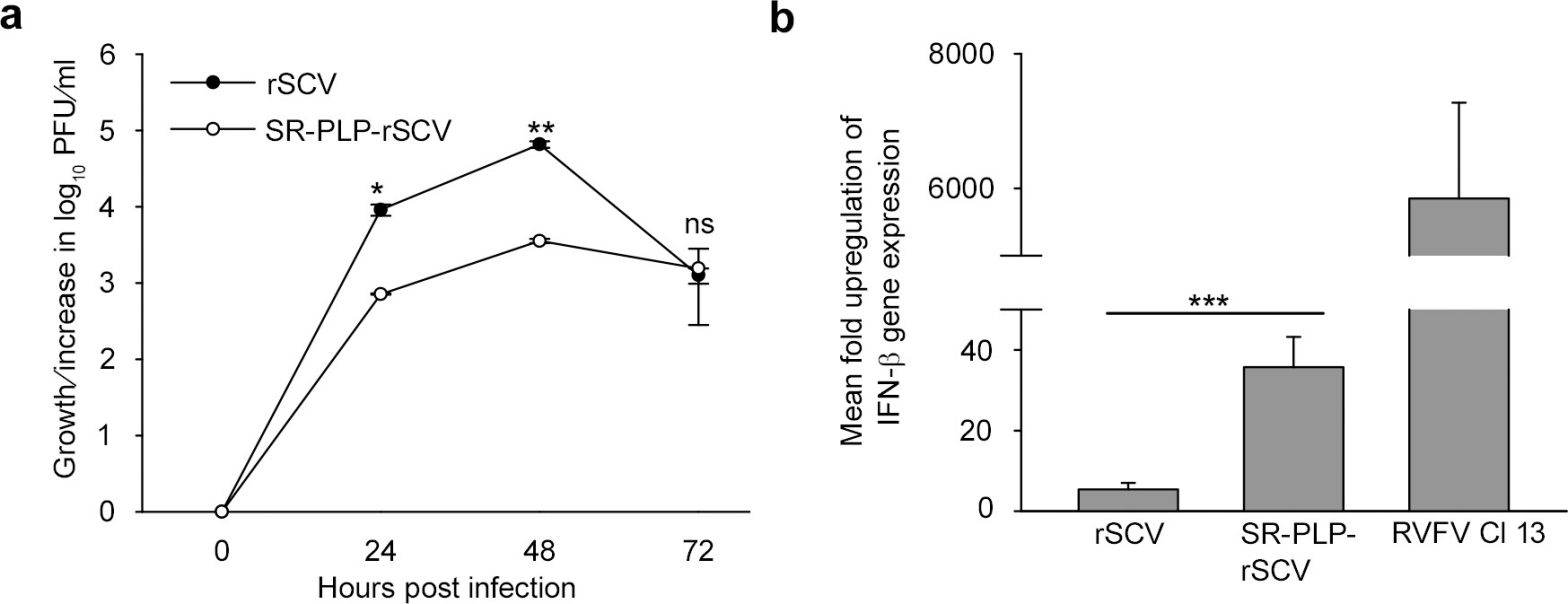
SR-PLP-rSCV grew less efficiently in IFN-competent, bat-derived lung cells. (a) For virus growth kinetics *Rhinolophus alcyone* lung cells, which expressed the SARS-CoV receptor human angiotensin converting enzyme (ACE)-2, were infected with rSCV and SR-PLP-rSCV at an MOI of 0.1. Supernatants were taken at 0, 24, 48 and 72 hpi, and viral replication was determined by a plaque titration assay. The experiment was performed in triplicate. Error bars indicate the standard deviations of the means. (b) RhiLu-hACE2 cells were infected with rSCV, SR-PLP-rSCV or IFN-inducing RVFV Cl 13 (control of *IFN-β* mRNA expression) at an MOI of 1. Total mRNA was extracted from cell lysates 24 hpi. *IFN-β* expression was determined using quantitative real-time PCR analysis. The mean fold change in the *IFN-β* expression was calculated using *β-actin* expression as a housekeeping/reference gene and the 2^−ΔΔCt^ analysis method [55]. The experiment was done in quadruplicates and repeated twice. One out of two experiments is shown. Statistical significance between the indicated groups was determined using a two-sided t test.

As ubiquitin (and its derivatives) are conserved in mammalian species, we expected that ubiquitin-dependent anti-IFN activities should be independent of the host species. As all members of the species SARS-CoV are carried by bats of the genus *Rhinolophus*, IFN-competent *Rhinolophus alcyone* lung epithelial cells (RhiLu) were made transgenic for the human ACE2 receptor (RhiLu-hACE2) by lentiviral transduction [56], and were infected with rSCV and SR-PLP-rSCV (**Fig 7A)**. In multistep growth curves, SR-PLP-rSCV grew to significantly lower titers compared to rSCV (13-fold lower at 24 hpi [p = 0.016] and 18-fold lower at 48 hpi [p = 0.005], respectively). Decreased viral growth of SR-PLP-rSCV in RhiLu-hACE2 bat cells may indicate more efficient IFN counteraction by rSCV compared to SR-PLP-rSCV, as in IFN-competent MA-104 cells. Remarkably, the growth differences were even more prominent in the bat cells compared to IFN competent primate cells (13-fold in RhiLu-hACE2 versus 3.6-fold in MA-104 cells at 24 hpi).

In order to investigate if the growth differences in bat cells were reflected by differences in IFN suppression, a quantitative real-time-PCR assay specific for the *Rhinolophus IFN-β* gene was established. To determine the specific *IFN-β* mRNA expression levels, RhiLu-hACE2 cells were infected with rSCV, SR-PLP-rSCV and, as IFN induction control, RVFV Cl 13 (MOI of 1). Total RNA was extracted from cell lysates at 24 hpi. To determine the fold-induction of the *IFN-β* mRNA expression levels, the 2^−ΔΔCt^ method was applied with *β-actin* as housekeeping gene [55]. In comparison to rSCV, SR-PLP-rSCV infection led to significantly higher *IFN-β* expression levels (6.6 fold at 24 hpi [p = 0.001] in a two-sided t test, **Fig 7B**).

In summary, these results suggest that the increased IFN sensitivity of SR-PLP is independent of the host cell species.

## Discussion

SARS-CoV variants exist across Europe in widespread *Rhinolophus* bat species. It is important to understand whether these viruses constitute a risk for human infection [57]. Host tropism is mainly thought to be determined by the spike protein, but studies have shown that CoV populations in natural reservoirs can contain a plethora of spike variants, including variants that can directly mediate entry into human cells [16, 20]. Because of widespread recombination, spike proteins can be exchanged between viral genetic lineages in the reservoir. The spike protein may not sufficiently represent the variability of virulence traits in the reservoir.

Here we studied a conserved viral function related to host interaction by focusing on PLP, a multifunctional protein that drives essential steps in the infection cycle including cleavage of the viral polyprotein as well as deubiquitination and deISGylation. While these latter functions interfere with molecules of the IFN pathway and cytokine production, it was unknown to what extent they contribute to the replication phenotype of SARS-CoV.

Our initial comparisons of PLP functions of epidemic and reservoir-associated virus were based on ectopically-expressed protein, confirming that essential properties such as cleavage of the viral polyprotein and sensitivity to a known inhibitor were almost identical between epidemic- and reservoir-derived PLP. However, overexpression assays cannot reflect the whole complexity of the viral life cycle including the timing and compartmentalization of functions. To detect more the subtle differences that we expected to occur between natural virus variants, we generated an infectious clone carrying the reservoir-derived PLP in an otherwise unmodified SARS-CoV backbone. Growth properties of the chimeric virus in IFN-deficient Vero cells confirmed that the reservoir-derived PLP effectively cleaved the viral polyprotein and supported recombinant virus replication kinetics comparable to wild type SARS-CoV. Only when grown in cells with a fully active IFN system, wild type SARS-CoV replicated better than the PLP-chimeric virus. The small but significant growth differences ranged between 2.6 (Calu-3) and 10.3-fold (HAE) depending on the time point post infection and the applied cell cultures. We assume that other viral proteins with IFN antagonistic functions, like protein 6 and 3b, might have compensated for the decreased immune-modulating functions of SR-PLP and PLP/M209R preventing a more pronounced growth difference [27]. Importantly, we showed that the effect was increased when artificial IFN was added to the cell cultures and was additionally accompanied by a more efficient blocking of *IFN-β* mRNA upregulation, obtaining further confirmation for the specificity of the PLP function within the IFN response.

We showed by studies on catalytically-inactivated PLP constructs that the anti-IFN function of the reservoir-derived SR-PLP depended on the protease function while SARS-CoV PLP exerted an additional, protease-independent activity in IFN evasion. The likely mechanism for this additional activity has been identified by *in-vitro* studies to involve binding of ubiquitin or ubiquitin-like modifiers [34, 54, 58]. Here we confirmed the functional link between DUB functions and IFN antagonism for the first time in a replicating CoV. Because the cytopathogenic nature of SARS-CoV infection prevented sensitive cell-based assays for DUB- and deISGylating functions, we relied on knowledge of structure-function relationships from a study on equine arteritis virus (EAV), an arterivirus related to CoVs [35]. In EAV PLP2, a ubiquitin-binding surface distinct from the protease active site was identified and mutagenized without causing a loss of protease processing. A definite link to IFN antagonism was established by engineering of a destabilizing mutation into the ubiquitin-binding surface in an EAV infectious clone, causing increased induction of IFN during replication. By introduction of a homologous mutation in our SARS-CoV infectious clone, we found an even clearer loss of IFN antagonism than with EAV. The choice of the mutation was informed by another study that defined the primary ubiquitin-binding surface in SARS-CoV PLP and found position M209 to critically interact with a conformational binding patch (I44, V70, L8) on ubiquitin but to not affect protease activity [54]. Concordantly, we found no signs of catalytic inactivation of the M209 mutant in Vero cells where it replicates like wild type virus, and a pattern of fully protease-dependent IFN antagonism like that of the reservoir-derived PLP. We conclude that ubiquitin binding constitutes an anti-IFN function of SARS-CoV PLP that is independent of the conserved protease function, is phenotypically relevant for replication level and immune evasion, and is variable among viral variants.

The IFN-related effects observed in the present study seem to be relevant as they resemble effects observed upon deletion of the 2’-O-methyltransferase activity, another essential replicase function that modifies IFN-recognition [59]. In that study, a D130A mutation in the nsp16 2’-O-methyltransferase domain replicated like wild type in Vero cells but caused about 10-fold lower replication in an IFN-competent human airway epithelial cell line. When engineered in a mouse-adapted backbone, the mutant was cleared faster from the lungs of infected mice, and caused significantly less weight loss. The present study did not use *in vivo* models to evaluate the consequences of the introduced mutations on replication. Mouse experiments may not reflect the role of PLP in host switching because ISG15 (expectably the most important ubiquitin-like modifier processed by SARS-CoV PLP [38]) is not conserved between humans, bats, and mice [60]. However, we can derive from the existence of SR-PLP in bats that the ubiquitin-related function of PLP is not essential for SARS-CoV replication in the natural host, and functional variants reflect natural diversity.

While it has been suggested that SARS-CoV may have gained virulence for humans during human-to-human passage and adaptation [12, 61], the differences in immune interaction observed in the present study are not dependent on the host cells used. Even in bat cells from the natural host of SARS-CoV, epidemic SARS-CoV has additional anti-IFN activity. Differential virulence via PLP may therefore have pre-existed within viral reservoir, rather than having evolved by positive selection upon adaptation within the human host. Therefore, we postulate that PLPs are a variable virulence trait among members of the species SARS-CoV that exist in natural reservoirs.

**Fig 8:**
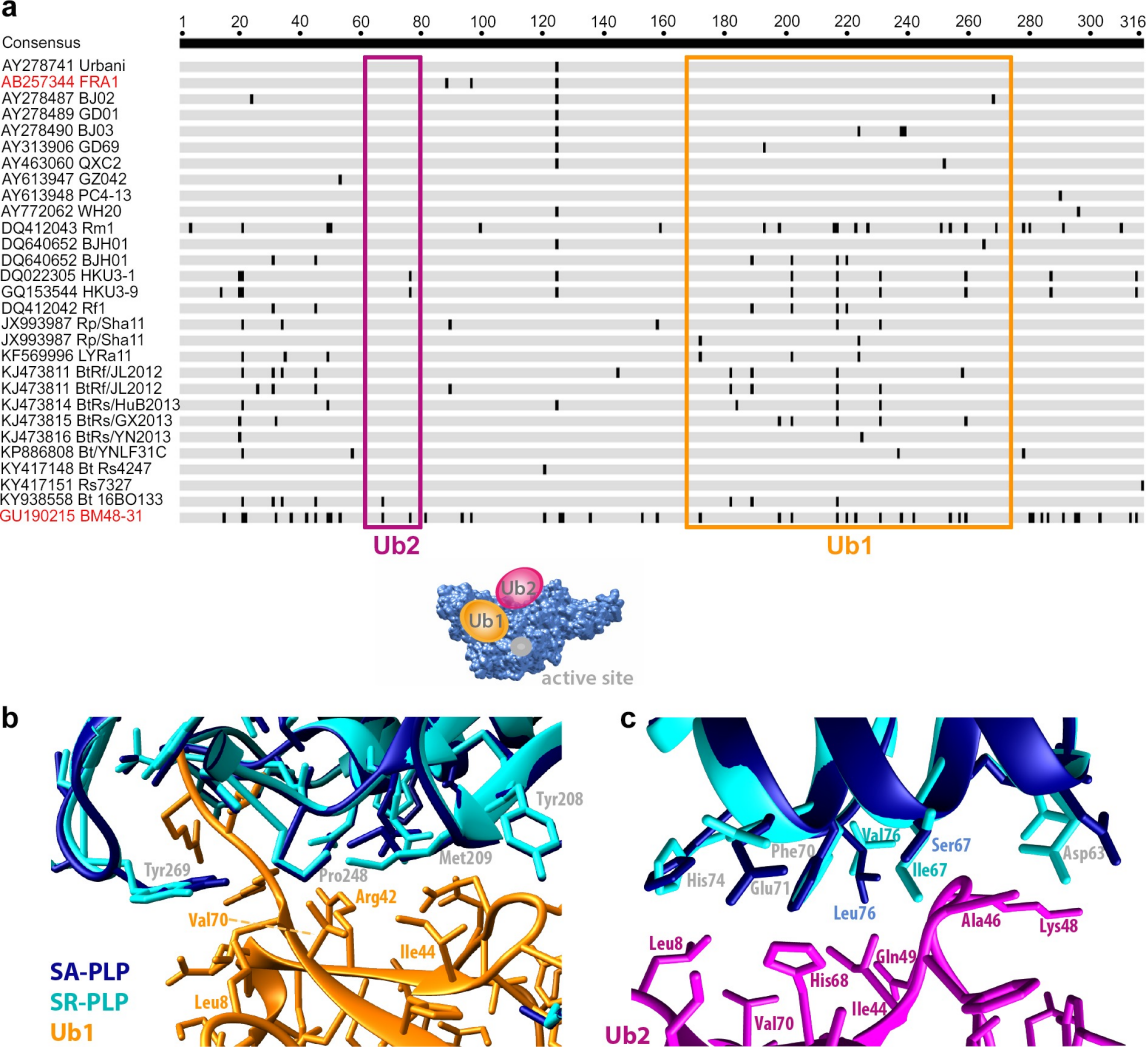
Variability in the PLPs of SARS-related CoVs found in the reservoirs in China and Europe. (a) Schematic representation of a PLP amino acid sequence alignment (315 amino acids) with European- and Chinese reservoir-associated SARS-related CoV strains which highlights the sequence diversity in the PLP domain. The alignment shows strains (represented as grey lines) with unique mutations (black vertical bars). The ubiquitin-binding surfaces are highlighted in orange (Ub1) and magenta (Ub2), respectively. The proposed localization of Ub1 and Ub2 is depicted in the cartoon below the alignment. SA- and SR-PLP, which were investigated in the presented study, are highlighted in red. Homology modelling for SR-PLP was done to compare the interaction surfaces between SA- and SR-PLP, respectively, and the two ubiquitin molecules of a K48-linked di-ubiquitin. Molecular graphics and analyzes were performed with the UCSF Chimera package [71] with the crystal structure of SARS-CoV PLP in complex with a K48-linked di-ubiquitin (PDB entry 5e6j) as template and the amino acid sequence of SR-PLP as a target. Putative amino acid contact points between SA- and SR-PLP and Ub1 (b) and Ub2 (c) highlight the conformational variability of both PLPs in ubiquitin-binding. The tertiary structure of SA-PLP is shown in dark blue, while the SR-PLP tertiary structure is shown in light blue.

Based on the PLP structure, there is huge potential for natural sequence variants to influence ubiquitin-binding. For instance, PLP has a second ubiquitin-binding site enabling a bi-dentate binding mechanism that may explain the PLP preference for di-ubiquitins and ISG15 [54]. Crystallization of PLP in complex with K48-linked di-ubiquitin identified the structure of the second binding site (Ub2), which seems to be more critical for K48-linked di-ubiquitin-binding than the primary binding surface [38]. The primary ubiquitin-binding surface (Ub1) is more critical for ISG15 binding [38, 60]. The SARS-CoV strains found in reservoirs in China and Europe show several variants at both ubiquitin-binding sites that could be studied for effects on replication level or other virulence traits (**Fig 8A**).

*In silico* modeling of the ubiquitin-binding sites of SA- and SR-PLP shows that sequence variations may even cause structural changes in- and adjacent to the binding sites (**Fig 8B and 8C**). The fact that, apart from our observed functional differences, there may also be structural changes between two closely related PLPs of a single virus species (the species “SARS-related CoV”) is particularly intriguing as such differences were previously only seen among CoVs that belong to different virus genera. The PLP of HCoV-NL63, for example, has similar protease-independent anti-IFN functions as the PLP of SARS-CoV whereas MERS-CoV PLP anti-IFN activity clearly depends on protease function [34, 37]. The existence of functional diversity in the reservoir makes sense in light of viral evolution and emergence. For instance, the capacity to modulate processing and substrate binding in separate domains of PLP opens the possibility for reservoir-associated CoVs to adjust fitness or virulence levels based on changes in host population structure. Increased virulence may confer increased transmission rates, but may come at the cost of host population decline. Based on our data it seems that the reservoir contains a considerable degree of functional variability that is based on highly conserved host functions such as the ubiquitin systems. Virus adaptation to the human host may not only be an evolved property at the beginning of virus emergence. Rather, the reservoir may contain pre-formed virulence traits that can be predicted by experiments informed by detailed knowledge of molecular virus functions, enabling new approaches to forecast emergence potential.

## Materials and methods

### Ethics statement

Fecal pellets of *Hipposideros* bats were provided by author Christian Drosten. All fecal samples were collected non-invasively by trained field biologists and stored at -80°C until analysis. In detail, bats were caught with mist nets, which were checked at intervals of 5 min. Captured bats were freed from nets immediately and put into cotton bags for several minutes. While being kept in bags, bats produced fecal pellets that were collected and transferred into RNAlater RNA stabilization solution (QIAGEN, Hilden, Germany). Procedures were previously described in [62], and were consistent with guidelines of the American society of mammalogists [63] for the use of wild mammals in research and national guidelines for the capture, handling, and care of bats. For all capturing, sampling and sample export, permission was obtained from the Wildlife Division of the Ministry of Lands, Forestry, and Mines in Ghana (permit no. CHRPE49/09; A04957) as described previously in [45]. Primary human airway epithelial cells were procured from patients who underwent surgical lung resection for any pulmonary disease and who gave informed consent. This was done in accordance with the ethical approval EKSG 11/044, EKSG 11/103 and KEK-BE 302/2015.

### Cells and viruses

HEK-293T (Friedemann Weber, University of Gießen) and MA-104 (Friedemann Weber, University of Gießen), Vero (Jindrich Cinatl, University of Frankfurt), Vero E6 (ATCC, ATCC CRL-1586), Calu-3 (ATCC HTB-55) and RhiLu-hACE2 (provided by author Marcel A. Müller [56]) cells were cultivated in Dulbecco’s modified Eagles medium (DMEM) supplemented with 10% fetal bovine serum (ThermoFisher Scientific), 1% penicillin/streptomycin, 1% non-essential amino acids, 1% L-glutamine and 1% sodium pyruvate in a 5% CO_2_ atmosphere at 37°C. HAE were generated and maintained as previously described [64]. RVFV Cl 13 was a kind gift from Friedemann Weber (University of Gießen). Infection experiments with rSCV were done under biosafety level 3 conditions with enhanced respiratory personal protection equipment.

### Plasmids

To ensure high level protein production of SA-, SR- and SO-PLPs in eukaryotic cells the codon-usage was optimized based on the human codon-usage frequency. In addition, potential splice sites and polyadenylation signal sequences were eliminated before the sequences were cloned into the eukaryotic expression plasmid pCAGGS along with a carboxy-terminal FLAG epitope tag. Regions of *SA-PLP* were PCR amplified from parental plasmid pPLpro1541-1855 [48]. *SR-* and *SO-PLPs* were synthesized by Geneart. Primers, used for cloning are listed in **S1 Table**. Site directed mutagenesis was done to change the catalytic cysteines into alanines (QuikChange Mutagenesis) and replace the ubiquitin-binding M209 residue to arginine (Gibson assembly, NEB) using the indicated primers below (**S1 Table**). The insertion of correct mutations was verified by DNA sequencing.

Nsp2/3-GFP, pCAGGS-HA-Ub, pISG15-myc and pRL-TK plasmids were kindly provided by Ralph S. Baric (University of North Carolina, USA), Adriano Marchese (Medical College of Wisconsin, USA, Min-Jung Kim (Pohang University, Republic of Korea) and Karl-Klaus Conzelmann (University of Munich, Germany). PcDNA3-Ube1L, pcDNA3-UbcH8 and pcDNA3-Herc5 were kind gifts from Robert M. Krug (University of Texas, USA). P125-Luc, pEF-BOS-MDA5-3xFLAG His10, GFP-IRF-3 and pCAGGS were previously described [65].

### (R)-N-(3-Acetamidobenzyl)-1-(1-(naphthalen-1-yl)ethyl)-piperidine-4-carboxamide (3e)

The synthesis and characterization of SA-PLP inhibitor 3e are described in [51].

### Generation of *SO-PLP* sequence

The *SO-PLP* sequence was obtained from a Hipposideros bat fecal sample (BUO2-B-F114) as previously described by Pfefferle et al. [45]. According to a nucleotide sequence alignment, containing related *PLP* gene sequences, primers were located to the most conserved regions within- and downstream of the *PLP* domain. Two different primer sets were applied in two successively performed PCR reactions. The nucleotide sequence information gained was used for the design of primers specifically targeting the *SO-PLP* sequence. The sequencing strategy and the primers are shown in **S2 and S3 Tables**.

### Chimeric SR-PLP-rSCV

SR-PLP-rSCV was constructed using the previously established cDNA clone [66]. This approach was based on bacterial artificial chromosomes (BAC) for keeping the full-length CoV cDNA stable. For the construction of the full-length infectious cDNA clone seven subclones (referred to as pA1, pA2, pB, pC, pD, pE and pF) covering the whole SARS-CoV genome were generated and assembled in a stepwise procedure. For the generation of SR-PLP-rSCV a chimeric SR-PLP subclone, named pBG-ABCD2, containing approximately one-half of the SARS-CoV genome and the desired *SR-PLP* replacement, was used. PBG-ABCD2 was joined with subclone pDEF into a full-length BAC cDNA clone and rescued as in [66]. SR-PLP-rSCV was sequenced to confirm the presence of *SR-PLP* and the absence of any further mutations with the following primers: primer for reverse transcription (Brev: 5’-TGAACCGCCACGCTGGCTAAACC-3’), sequencing primers (B4622F: 5’-CTTAAAGCTCCTGCCGTAGTG-3’, BG4792F: 5’-TATTAAGGTGTTCACAACTGTAG-3’, BG5631F: 5’-AAATTGATGGTGCTCTCTTGAC-3’)

### Virus infection

Cells were seeded at a concentration of 3.5x10^5^ cells/ml. After 24 h, virus stocks were diluted in serum-free medium according to the desired MOI. For virus adsorption 200 μl (24-well) or 1 ml (6-well) of virus master mix was added to the cells and incubated for 1 h at 37°C. After 1 h, the virus dilutions were removed and the wells were washed twice with 1x PBS and refilled with supplemented DMEM. Supernatants were taken at the indicated time points and studied further.

For infection of HAE air liquid interface cultures, the apical surface was washed twice with 200 μl Hank’s balanced salt solution (HBSS) to remove mucus. Virus stocks were diluted in HBSS and HAE were infected with an absolute infectious dose of 40,000 PFU. Cells were incubated for 1.5 h at 37°C in a 5% CO_2_ atmosphere with 95% humidity. After adsorption, virus dilutions were removed and the wells were washed three times with 200 μl HBSS. Samples were taken at the indicated time points by applying 200 μl HBSS to the apical surface 10 minutes prior to the actual time points. Basolateral medium was exchanged at 48 hpi.

### *Trans*-cleavage assay

Assessment of protease activity was conducted by a *trans*-cleavage assay. 2x10^5^ cells/ml were seeded in a 12-well plate 24 h prior to transfection. 300 ng of *PLP*-encoding plasmids were coexpressed with 25 ng of SARS-CoV nsp2/nsp3-GFP substrate [49]. After 16 h, cells were lysed with 100 μl lysis buffer (20 mM Tris [pH 7.5], 150 mM NaCl, 1 mM EGTA, 1 mM EDTA, 1% Triton X-100, 12.5 mM Na pyrophosphate, 1 mM β-glycerophosphate, 1 mM Na ortho-vanadate, 1 mg/ml leupeptin, 1 mM PMSF) and lysates were separated by SDS-PAGE using a semi-dry transfer blotter. After protein transfer, the membrane was blocked by 5% dried milk in TBST buffer (0.9% NaCl, 10 mM Tris-HCl [pH 7.5], 0.1% Tween 20) for 1 h at room temperature. The membrane was incubated with a rabbit-antibody directed against the GFP-epitope tag (ThermoFisher Scientific). The membrane was washed three times for 15 min in TBST buffer. Next, the membrane was incubated with HRP-coupled donkey anti-rabbit secondary antibody (SouthernBiotech). After 1 h, the membrane was washed three times for 15 min in TBST buffer. To confirm *PLP* expression, the membrane was probed with mouse anti-FLAG (Sigma-Aldrich) followed by goat anti-mouse HRP-coupled (SouthernBiotech) antibodies. Mouse anti-β-actin (Sigma-Aldrich) was used to detect host cell proteins as a loading control. Secondary detection was performed using goat anti-mouse HRP antibody (SouthernBiotech).

### Biosensor assay

The assay was performed using HEK-293T cells in black 96-well plates with clear bottom. Cells were transfected with 37.5 ng pGlo-30F-RLKGG [50] and 50 ng *PLP*-expressing plasmids using Fugene HD (Promega) according to the manufacturer’s instructions. At 14 hours post transfection (hpt), cells were equilibrated with GloSensor reagent (Promega). For inhibitor quantification, cells were equilibrated at 13 hpt with GloSensor reagent as indicated above and at 14 hpt diluted inhibitor or DMSO was added. Luminescence was measured after 1 h for the following 6 h. For data analysis, the fold luciferase induction was calculated independently for each PLP by calibrating the values, obtained for every time point to the respective starting values.

### DUB activity assay

HEK-293T cells (2x10^5^ cells/ml) were transfected in the 12-well format with 300 ng of HA-ubiquitin (HA-Ub) and 50, 100 or 200 ng *PLP*-expressing plasmids using TransIT-LT1 transfection reagent (Mirus). At 18 hpt, cells were treated with 100 μl of lysis buffer. Proteins were separated by SDS-PAGE and analyzed by Western blotting as described above. Western blot analysis was done using mouse anti-HA serum (Covance) and goat anti-mouse HRP (SouthernBiotech) antibodies.

### DeISGylation assay

HEK-293T cells were transfected with plasmids encoding for myc-epitope tagged *ISG15* (250 ng of pISG15-myc) and its conjugation machinery, comprising a set of three ligases (UbcH8/125 ng pUbcH8, Ube1L/125 ng pUbe1L and Herc5/125 ng pHerc5) as in [40]. *PLP*-encoding plasmids were cotransfected in amounts of 100 ng. After 18 h, cells were lysed and separated by SDS-PAGE as indicated above. The extent of ISGylated cellular proteins was analyzed by Western blotting using mouse anti-myc (MBL Life science) and goat anti-mouse HRP (SouthernBiotech) antibodies.

### Dual luciferase assay

2x10^5^ HEK-293T cells/ml were transfected with plasmids encoding *Renilla* (RL) and *Firefly* (FF) luciferases. The *FF* luciferase gene was under control of an IFN-β promoter (p125-luc). *RL* luciferase gene was cloned behind a herpes simplex thymidine-kinase (TK) promoter. IFN-β promoter activation was triggered by the overexpression of the cellular *MDA5*, which led to an auto-activation of the IFN pathway [67]. Transfection of DNA plasmids was done with Fugene HD (Promega) according to the manufacturer’s instructions. Plasmid amounts are given for the 24-well plate format and are as follows: pRL-TK (5 ng), p125-luc (250 ng), pEF-BOS-MDA5-3xFLAG His10 (100 ng) and 1 to 50 ng of *PLP*-expressing plasmids. Cells were lysed at 17 hpt. 20 μl of luciferase-containing lysate was transferred to an opaque, white 96-well microtiter plate and used for assessment of luciferases activity by a bioluminescence detection reader.

### Translocation assay

The IRF-3 translocation assay was performed as described elsewhere [65]. Briefly, MA-104 cells were transfected with plasmids encoding 250 ng GFP-IRF-3 and either EV or 1000 ng of FLAG-tagged *PLP*-expressing plasmids using Fugene HD (Promega). To activate the IFN pathway, cells were infected with RVFV Cl 13 and an MOI of 5 at 17 hpt. At 8 hpi cells were fixed with 4% paraformaldehyde and permeabilized with 0.1% TritonX-100. Immunofluorescence analysis was done as in [68]. Samples transfected with EV were treated with anti-RVFV mouse serum (Friedemann Weber, University of Gießen, [53]) and goat anti-mouse Cy3 (Dianova GmbH) secondary antibody. *PLP*-expressing cells were stained with mouse anti-FLAG (Sigma-Aldrich) and goat anti-mouse Cy3 (Dianova GmbH) antibodies. Samples were analyzed by a fluorescence microscope (Zeiss). Depending on the number of transfected cells, at least three images were taken, and the number of cells double-positive for GFP and FLAG was divided by the number of cells showing IRF-3 nuclear translocations.

### Plaque titration assay

3.5x10^5^ VeroE6 cells/ml were seeded in a 24-well plate 24 h prior to infection. A 1:10 serial dilution of samples was generated, 200 μl dilution was added to the cells and incubated for 1 h at 37°C for adsorption. After virus samples were removed, 500 μl overlay (2.4% avicel diluted in 2x DMEM) was added to each well. The overlay was discarded at 3 days post infection (dpi) and cells were fixed for 30 min in 6% formaldehyde. The cells were washed once with 1x PBS and stained with crystal violet working solution for 15 min. Plaque forming units were determined from at least two dilutions for which distinct plaques were detectable.

### Real-time RT-PCR assays

Viral RNA was extracted with the NucleoSpin RNA virus isolation kit (Macherey-Nagel) after the manufacturer’s instructions. 1 μl of viral RNA was applied to each reaction. Quantification of genomic SARS-CoV RNA was done using the SuperScript III one-step reverse transcriptase-PCR system (Invitrogen) with the Platinum Taq DNA polymerase according to the manufacturer’s recommendations and these primers (SARS-F: 5’-CCCGCGAAGAAGCTATTCG-3’, SARS-P: Fam-5’-ACGTTCGTGCGTGGATTGGCTTTG-3’-BHQ, SARS-R: 5’-AGTTGCATGACAGCCCTCTACA-3’). An established SARS-CoV standard curve was used in each run [69] to quantify RNA copies per ml.

To analyze *IFN-β* expression in Calu-3 and *Rhinolophus alcyone* cells (provided by authors Christian Drosten and Marcel A. Müller), mRNA was extracted with the NucleoSpin RNA isolation kit (Macherey-Nagel). Real-time PCR was done as described previously [70] using the following primers: Hu-IFNB1-F: 5’-AGGATTCTGCATTACCTGAAGG-3’, Hu-IFNB1-P: Fam-5’- TCCACTCTGACTATGGTCCAGGCA-3’-ZEN and Hu-IFNB1-R: 5’-GGCTAGGAGATCTTCAGTTTCG-3’ (Calu-3) and Rhi-IFNB1-F: 5’-AAATAATGGAGGAGGAAAACTTCAC-3’, Rhi-IFNB1-P: Fam-5’-CGAAACATGAGCACGCTGCACCTG-3’-BHQ and Rhi-IFNB1-R: 5’- CGCCTGATCCTTAGGTAGTAATTCT-3’ (*Rhinolophus alcyone*). To determine the induction of *IFN-β* relative to *TBP* (Calu-3) and *β-actin* (*Rhinolophus alcyone*) the 2^−ΔΔCt^ method was applied [55].

### Alignments, phylogenetic analysis, similarity/identity calculations

Phylogenetic analysis was performed using the neighbor joining algorithm in Geneious 9.1.5 under the assumption of a Tamura-Nei genetic distance model. Alignments were done based on the amino acid codes by the BLOSUM62 algorithm in the Geneious 9.1.5 software package. The identities and similarities of the listed proteins were calculated from the amino acid alignment using the BLOSUM62 substitution matrix with a threshold of 1.

### Accession numbers

SO-PLP: MG916963

## Supporting information

S1 Fig Analysis of SR-PLP splicing products by Western Blot analysis. HEK-293T cells were transfected with a plasmid expressing non-optimized SR-PLP. For detection of potential splicing products the amino-terminus contained a Flag- and the carboxy-terminus contained an HA-tag. Protein expression was confirmed by Western Blot analysis using Flag- or HA-specific antibodies produced in mouse, respectively. Secondary detection was done using anti-mouse antibodies coupled with horseradish peroxidase, which were produced in goat.
(TIF)

S2 Fig Comparative inhibition of rSCV and chimeric SR-PLP-rSCV by compound 3e. The number of viable cells after inhibitor treatment was determined by a luminescence-based cell viability assay. Therefore, Vero cells were seeded in an opaque 96-well plate and after 20 h, cells were incubated with either DMSO or a serially diluted compound 3e using DMEM as diluent. After 24 h, 100 μl of detection reagent was added and incubated for another 15 min. Emitted luminescence was recorded by a detection reader with an integration time of 1s.
(TIF)

S1 Table Primers for cloning of the constructs used for heterologous expression analyzes. ^a^Underlined nucleotides were added for cloning purposes. ^b^FLAG tag is highlighted in bold. For: Forward primer; Rev: Reverse primer.
(DOCX)

S2 Table Amplification strategy for the generation of *SO-PLP* sequence from bat fecal sample BUO2-B-F114. SSIII one step: SuperScript III one-step RT-PCR system with Platinum Taq DNA polymerase.
(DOCX)

S3 Table Primers for the amplification and sequencing of *SO-PLP*. For: Forward primer; Rev: Reverse primer. The single-letter code system for degenerated bases established by the international union of pure and applied chemistry (IUPAC) was used for definition [72].
(DOCX)

## References

[1] Vijgen L, Keyaerts E, Lemey P, Maes P, Van Reeth K, Nauwynck H, et al Evolutionary history of the closely related group 2 coronaviruses: porcine hemagglutinating encephalomyelitis virus, bovine coronavirus, and human coronavirus OC43. J Virol. 2006;80(14):7270–4. 10.1128/JVI.02675-05 16809333 PMC1489060

[2] Vijgen L, Keyaerts E, Moes E, Thoelen I, Wollants E, Lemey P, et al Complete genomic sequence of human coronavirus OC43: molecular clock analysis suggests a relatively recent zoonotic coronavirus transmission event. J Virol. 2005;79(3):1595–604. 10.1128/JVI.79.3.1595-1604.2005 15650185 PMC544107

[3] Han MG, Cheon DS, Zhang X, Saif LJ. Cross-protection against a human enteric coronavirus and a virulent bovine enteric coronavirus in gnotobiotic calves. J Virol. 2006;80(24):12350–6. 10.1128/JVI.00402-06 16971444 PMC1676286

[4] Corman VM, Eckerle I, Memish ZA, Liljander AM, Dijkman R, Jonsdottir H, et al Link of a ubiquitous human coronavirus to dromedary camels. Proc Natl Acad Sci U S A. 2016;113(35):9864–9. 10.1073/pnas.1604472113 27528677 PMC5024591

[5] Zaki AM, van Boheemen S, Bestebroer TM, Osterhaus AD, Fouchier RA. Isolation of a novel coronavirus from a man with pneumonia in Saudi Arabia. N Engl J Med. 2012;367(19):1814–20. 10.1056/NEJMoa1211721 23075143

[6] Liljander A, Meyer B, Jores J, Muller MA, Lattwein E, Njeru I, et al MERS-CoV Antibodies in Humans, Africa, 2013–2014. Emerg Infect Dis. 2016;22(6):1086–9. 10.3201/eid2206.160064 27071076 PMC4880087

[7] Seong MW, Kim SY, Corman VM, Kim TS, Cho SI, Kim MJ, et al Microevolution of Outbreak-Associated Middle East Respiratory Syndrome Coronavirus, South Korea, 2015. Emerg Infect Dis. 2016;22(2):327–30. 10.3201/eid2202.151700 26814649 PMC4734539

[8] Muller MA, Meyer B, Corman VM, Al-Masri M, Turkestani A, Ritz D, et al Presence of Middle East respiratory syndrome coronavirus antibodies in Saudi Arabia: a nationwide, cross-sectional, serological study. Lancet Infect Dis. 2015;15(6):629. 10.1016/S1473-3099(15)00029-8 26008827

[9] Reusken CB, Haagmans BL, Muller MA, Gutierrez C, Godeke GJ, Meyer B, et al Middle East respiratory syndrome coronavirus neutralising serum antibodies in dromedary camels: a comparative serological study. Lancet Infect Dis. 2013;13(10):859–66. 10.1016/S1473-3099(13)70164-6 23933067 PMC7106530

[10] WHO. WHO Summary of probable SARS cases with onset of illness from 1 November 2002 to 31 July 2003. (Based on data as of the 31 December 2003.) 2004;Available from: http://www.who.int/csr/sars/country/table2004_04_21/en/.

[11] Peiris JS, Yuen KY, Osterhaus AD, Stohr K. The severe acute respiratory syndrome. N Engl J Med. 2003;349(25):2431–41. 10.1056/NEJMra032498 14681510

[12] Chinese SMEC. Molecular evolution of the SARS coronavirus during the course of the SARS epidemic in China. Science. 2004;303(5664):1666–9. 10.1126/science.1092002 14752165

[13] Che XY, Di B, Zhao GP, Wang YD, Qiu LW, Hao W, et al A patient with asymptomatic severe acute respiratory syndrome (SARS) and antigenemia from the 2003–2004 community outbreak of SARS in Guangzhou, China. Clin Infect Dis. 2006;43(1):e1–5. 10.1086/504943 16758408 PMC7108013

[14] Becker MM, Graham RL, Donaldson EF, Rockx B, Sims AC, Sheahan T, et al Synthetic recombinant bat SARS-like coronavirus is infectious in cultured cells and in mice. Proc Natl Acad Sci U S A. 2008;105(50):19944–9. 10.1073/pnas.0808116105 19036930 PMC2588415

[15] Menachery VD, Yount BL Jr., Debbink K, Agnihothram S, Gralinski LE, Plante JA, et al A SARS-like cluster of circulating bat coronaviruses shows potential for human emergence. Nat Med. 2015;21(12):1508–13. 10.1038/nm.3985 26552008 PMC4797993

[16] Hu B, Zeng LP, Yang XL, Ge XY, Zhang W, Li B, et al Discovery of a rich gene pool of bat SARS-related coronaviruses provides new insights into the origin of SARS coronavirus. PLoS Pathog. 2017;13(11):e1006698 10.1371/journal.ppat.1006698 29190287 PMC5708621

[17] Cyranoski D. Bat cave solves mystery of deadly SARS virus—and suggests new outbreak could occur. Nature. 2017;552(7683):15–6. 10.1038/d41586-017-07766-9 29219990

[18] Wang L, Fu S, Cao Y, Zhang H, Feng Y, Yang W, et al Discovery and genetic analysis of novel coronaviruses in least horseshoe bats in southwestern China. Emerg Microbes Infect. 2017;6(3):e14 10.1038/emi.2016.140 28352124 PMC5378919

[19] Graham RL, Baric RS. Recombination, reservoirs, and the modular spike: mechanisms of coronavirus cross-species transmission. J Virol. 2010;84(7):3134–46. 10.1128/JVI.01394-09 19906932 PMC2838128

[20] Ge XY, Li JL, Yang XL, Chmura AA, Zhu G, Epstein JH, et al Isolation and characterization of a bat SARS-like coronavirus that uses the ACE2 receptor. Nature. 2013;503(7477):535–8. 10.1038/nature12711 24172901 PMC5389864

[21] Ren W, Qu X, Li W, Han Z, Yu M, Zhou P, et al Difference in receptor usage between severe acute respiratory syndrome (SARS) coronavirus and SARS-like coronavirus of bat origin. J Virol. 2008;82(4):1899–907. 10.1128/JVI.01085-07 18077725 PMC2258702

[22] Drosten C. Virus ecology: a gap between detection and prediction. Emerging Microbes & Infections. 2013;2:e31. 26038466 10.1038/emi.2013.25 PMC3675401

[23] Haller O, Kochs G, Weber F. The interferon response circuit: induction and suppression by pathogenic viruses. Virology. 2006;344(1):119–30. 10.1016/j.virol.2005.09.024 16364743 PMC7125643

[24] Randall RE, Goodbourn S. Interferons and viruses: an interplay between induction, signalling, antiviral responses and virus countermeasures. J Gen Virol. 2008;89(Pt 1):1–47. 10.1099/vir.0.83391-0 18089727

[25] Roth-Cross JK, Bender SJ, Weiss SR. Murine coronavirus mouse hepatitis virus is recognized by MDA5 and induces type I interferon in brain macrophages/microglia. J Virol. 2008;82(20):9829–38. 10.1128/JVI.01199-08 18667505 PMC2566260

[26] Zhong B, Yang Y, Li S, Wang YY, Li Y, Diao F, et al The adaptor protein MITA links virus-sensing receptors to IRF3 transcription factor activation. Immunity. 2008;29(4):538–50. 10.1016/j.immuni.2008.09.003 18818105

[27] Kopecky-Bromberg SA, Martinez-Sobrido L, Frieman M, Baric RA, Palese P. Severe acute respiratory syndrome coronavirus open reading frame (ORF) 3b, ORF 6, and nucleocapsid proteins function as interferon antagonists. J Virol. 2007;81(2):548–57. 10.1128/JVI.01782-06 17108024 PMC1797484

[28] Tohya Y, Narayanan K, Kamitani W, Huang C, Lokugamage K, Makino S. Suppression of host gene expression by nsp1 proteins of group 2 bat coronaviruses. J Virol. 2009;83(10):5282–8. 10.1128/JVI.02485-08 19264783 PMC2682096

[29] Mielech AM, Chen Y, Mesecar AD, Baker SC. Nidovirus papain-like proteases: multifunctional enzymes with protease, deubiquitinating and deISGylating activities. Virus Res. 2014;194:184–90. 10.1016/j.virusres.2014.01.025 24512893 PMC4125544

[30] Chen X, Yang X, Zheng Y, Yang Y, Xing Y, Chen Z. SARS coronavirus papain-like protease inhibits the type I interferon signaling pathway through interaction with the STING-TRAF3-TBK1 complex. Protein Cell. 2014;5(5):369–81. 10.1007/s13238-014-0026-3 24622840 PMC3996160

[31] Sun L, Xing Y, Chen X, Zheng Y, Yang Y, Nichols DB, et al Coronavirus papain-like proteases negatively regulate antiviral innate immune response through disruption of STING-mediated signaling. PLoS One. 2012;7(2):e30802 10.1371/journal.pone.0030802 22312431 PMC3270028

[32] Komander D, Rape M. The ubiquitin code. Annu Rev Biochem. 2012;81:203–29. 10.1146/annurev-biochem-060310-170328 22524316

[33] Nicholson B, Leach CA, Goldenberg SJ, Francis DM, Kodrasov MP, Tian X, et al Characterization of ubiquitin and ubiquitin-like-protein isopeptidase activities. Protein Sci. 2008;17(6):1035–43. 10.1110/ps.083450408 18424514 PMC2386736

[34] Clementz MA, Chen Z, Banach BS, Wang Y, Sun L, Ratia K, et al Deubiquitinating and interferon antagonism activities of coronavirus papain-like proteases. J Virol. 2010;84(9):4619–29. 10.1128/JVI.02406-09 20181693 PMC2863753

[35] van Kasteren PB, Bailey-Elkin BA, James TW, Ninaber DK, Beugeling C, Khajehpour M, et al Deubiquitinase function of arterivirus papain-like protease 2 suppresses the innate immune response in infected host cells. Proc Natl Acad Sci U S A. 2013;110(9):E838–47. 10.1073/pnas.1218464110 23401522 PMC3587229

[36] Frias-Staheli N, Giannakopoulos NV, Kikkert M, Taylor SL, Bridgen A, Paragas J, et al Ovarian Tumor Domain-Containing Viral Proteases Evade Ubiquitin- and ISG15-Dependent Innate Immune Responses. Cell Host & Microbe. 2007;2(6):404–16. 18078692 10.1016/j.chom.2007.09.014 PMC2184509

[37] Baez-Santos YM, Mielech AM, Deng X, Baker S, Mesecar AD. Catalytic function and substrate specificity of the papain-like protease domain of nsp3 from the Middle East respiratory syndrome coronavirus. J Virol. 2014;88(21):12511–27. 10.1128/JVI.01294-14 25142582 PMC4248884

[38] Bekes M, van der Heden van Noort GJ, Ekkebus R, Ovaa H, Huang TT, Lima CD. Recognition of Lys48-Linked Di-ubiquitin and Deubiquitinating Activities of the SARS Coronavirus Papain-like Protease. Mol Cell. 2016;62(4):572–85. 10.1016/j.molcel.2016.04.016 27203180 PMC4875570

[39] Clasman JR, Baez-Santos YM, Mettelman RC, O'Brien A, Baker SC, Mesecar AD. X-ray Structure and Enzymatic Activity Profile of a Core Papain-like Protease of MERS Coronavirus with utility for structure-based drug design. Sci Rep. 2017;7:40292 10.1038/srep40292 28079137 PMC5228125

[40] Mielech AM, Kilianski A, Baez-Santos YM, Mesecar AD, Baker SC. MERS-CoV papain-like protease has deISGylating and deubiquitinating activities. Virology. 2014;450–451:64–70. 10.1016/j.virol.2013.11.040 24503068 PMC3957432

[41] Bailey-Elkin BA, Knaap RC, Johnson GG, Dalebout TJ, Ninaber DK, van Kasteren PB, et al Crystal structure of the Middle East respiratory syndrome coronavirus (MERS-CoV) papain-like protease bound to ubiquitin facilitates targeted disruption of deubiquitinating activity to demonstrate its role in innate immune suppression. J Biol Chem. 2014;289(50):34667–82. 10.1074/jbc.M114.609644 25320088 PMC4263872

[42] Kindler E, Jonsdottir HR, Muth D, Hamming OJ, Hartmann R, Rodriguez R, et al Efficient replication of the novel human betacoronavirus EMC on primary human epithelium highlights its zoonotic potential. MBio. 2013;4(1):e00611–12. 10.1128/mBio.00611-12 23422412 PMC3573664

[43] Zielecki F, Weber M, Eickmann M, Spiegelberg L, Zaki AM, Matrosovich M, et al Human cell tropism and innate immune system interactions of human respiratory coronavirus EMC compared to those of severe acute respiratory syndrome coronavirus. J Virol. 2013;87(9):5300–4. 10.1128/JVI.03496-12 23449793 PMC3624328

[44] Drexler JF, Gloza-Rausch F, Glende J, Corman VM, Muth D, Goettsche M, et al Genomic characterization of severe acute respiratory syndrome-related coronavirus in European bats and classification of coronaviruses based on partial RNA-dependent RNA polymerase gene sequences. J Virol. 2010;84(21):11336–49. 10.1128/JVI.00650-10 20686038 PMC2953168

[45] Pfefferle S, Oppong S, Drexler JF, Gloza-Rausch F, Ipsen A, Seebens A, et al Distant relatives of severe acute respiratory syndrome coronavirus and close relatives of human coronavirus 229E in bats, Ghana. Emerg Infect Dis. 2009;15(9):1377–84. 10.3201/eid1509.090224 19788804 PMC2819850

[46] Harcourt BH, Jukneliene D, Kanjanahaluethai A, Bechill J, Severson KM, Smith CM, et al Identification of severe acute respiratory syndrome coronavirus replicase products and characterization of papain-like protease activity. J Virol. 2004;78(24):13600–12. 10.1128/JVI.78.24.13600-13612.2004 15564471 PMC533933

[47] Rawlings ND, Barrett AJ. [32] Families of cysteine peptidases Methods in Enzymology. 244: Academic Press; 1994 p. 461–86. 7845226 10.1016/0076-6879(94)44034-4 PMC7172846

[48] Barretto N, Jukneliene D, Ratia K, Chen Z, Mesecar AD, Baker SC. The papain-like protease of severe acute respiratory syndrome coronavirus has deubiquitinating activity. J Virol. 2005;79(24):15189–98. 10.1128/JVI.79.24.15189-15198.2005 16306590 PMC1316023

[49] Frieman M, Ratia K, Johnston RE, Mesecar AD, Baric RS. Severe acute respiratory syndrome coronavirus papain-like protease ubiquitin-like domain and catalytic domain regulate antagonism of IRF3 and NF-kappaB signaling. J Virol. 2009;83(13):6689–705. 10.1128/JVI.02220-08 19369340 PMC2698564

[50] Kilianski A, Mielech AM, Deng X, Baker SC. Assessing activity and inhibition of Middle East respiratory syndrome coronavirus papain-like and 3C-like proteases using luciferase-based biosensors. J Virol. 2013;87(21):11955–62. 10.1128/JVI.02105-13 23986593 PMC3807373

[51] Baez-Santos YM, Barraza SJ, Wilson MW, Agius MP, Mielech AM, Davis NM, et al X-ray structural and biological evaluation of a series of potent and highly selective inhibitors of human coronavirus papain-like proteases. J Med Chem. 2014;57(6):2393–412. 10.1021/jm401712t 24568342 PMC3983375

[52] Kuri T, Habjan M, Penski N, Weber F. Species-independent bioassay for sensitive quantification of antiviral type I interferons. Virol J. 2010;7:50 10.1186/1743-422X-7-50 20187932 PMC2846901

[53] Habjan M, Andersson I, Klingstrom J, Schumann M, Martin A, Zimmermann P, et al Processing of genome 5' termini as a strategy of negative-strand RNA viruses to avoid RIG-I-dependent interferon induction. PLoS One. 2008;3(4):e2032 10.1371/journal.pone.0002032 18446221 PMC2323571

[54] Ratia K, Kilianski A, Baez-Santos YM, Baker SC, Mesecar A. Structural Basis for the Ubiquitin-Linkage Specificity and deISGylating activity of SARS-CoV papain-like protease. PLoS Pathog. 2014;10(5):e1004113 10.1371/journal.ppat.1004113 24854014 PMC4031219

[55] Livak KJ, Schmittgen TD. Analysis of relative gene expression data using real-time quantitative PCR and the 2(-Delta Delta C(T)) Method. Methods. 2001;25(4):402–8. 10.1006/meth.2001.1262 11846609

[56] Muller MA, Raj VS, Muth D, Meyer B, Kallies S, Smits SL, et al Human coronavirus EMC does not require the SARS-coronavirus receptor and maintains broad replicative capability in mammalian cell lines. MBio. 2012;3(6). 10.1128/mBio.00515-12 PMC3520110 23232719

[57] Drexler JF, Corman VM, Drosten C. Ecology, evolution and classification of bat coronaviruses in the aftermath of SARS. Antiviral Res. 2014;101:45–56. 10.1016/j.antiviral.2013.10.013 24184128 PMC7113851

[58] Devaraj SG, Wang N, Chen Z, Chen Z, Tseng M, Barretto N, et al Regulation of IRF-3-dependent innate immunity by the papain-like protease domain of the severe acute respiratory syndrome coronavirus. J Biol Chem. 2007;282(44):32208–21. 10.1074/jbc.M704870200 17761676 PMC2756044

[59] Menachery VD, Yount BL Jr., Josset L, Gralinski LE, Scobey T, Agnihothram S, et al Attenuation and restoration of severe acute respiratory syndrome coronavirus mutant lacking 2'-o-methyltransferase activity. J Virol. 2014;88(8):4251–64. 10.1128/JVI.03571-13 24478444 PMC3993736

[60] Daczkowski CM, Dzimianski JV, Clasman JR, Goodwin O, Mesecar AD, Pegan SD. Structural Insights into the Interaction of Coronavirus Papain-Like Proteases and Interferon-Stimulated Gene Product 15 from Different Species. J Mol Biol. 2017;429(11):1661–83. 10.1016/j.jmb.2017.04.011 28438633 PMC5634334

[61] Song HD, Tu CC, Zhang GW, Wang SY, Zheng K, Lei LC, et al Cross-host evolution of severe acute respiratory syndrome coronavirus in palm civet and human. Proc Natl Acad Sci U S A. 2005;102(7):2430–5. 10.1073/pnas.0409608102 15695582 PMC548959

[62] Gloza-Rausch F, Ipsen A, Seebens A, Gottsche M, Panning M, Drexler JF, et al Detection and prevalence patterns of group I coronaviruses in bats, northern Germany. Emerg Infect Dis. 2008;14(4):626–31. 10.3201/eid1404.071439 18400147 PMC2570906

[63] Sikes RS, Gannon WL, mammalogists AcaucotAso. Guidelines of the American Society of Mammalogists for the use of wild mammals in research. Journal of Mammalogy. 2011;92(1):235–53. 10.1093/jmammal/gyw078 PMC5909806 29692469

[64] Dijkman R, Koekkoek SM, Molenkamp R, Schildgen O, van der Hoek L. Human bocavirus can be cultured in differentiated human airway epithelial cells. J Virol. 2009;83(15):7739–48. 10.1128/JVI.00614-09 19474096 PMC2708629

[65] Niemeyer D, Zillinger T, Muth D, Zielecki F, Horvath G, Suliman T, et al Middle East respiratory syndrome coronavirus accessory protein 4a is a type I interferon antagonist. J Virol. 2013;87(22):12489–95. 10.1128/JVI.01845-13 24027320 PMC3807936

[66] Pfefferle S, Krahling V, Ditt V, Grywna K, Muhlberger E, Drosten C. Reverse genetic characterization of the natural genomic deletion in SARS-Coronavirus strain Frankfurt-1 open reading frame 7b reveals an attenuating function of the 7b protein in-vitro and in-vivo. Virol J. 2009;6:131 10.1186/1743-422X-6-131 19698190 PMC2739521

[67] Childs K, Randall R, Goodbourn S. Paramyxovirus V proteins interact with the RNA Helicase LGP2 to inhibit RIG-I-dependent interferon induction. J Virol. 2012;86(7):3411–21. 10.1128/JVI.06405-11 22301134 PMC3302505

[68] Corman VM, Muller MA, Costabel U, Timm J, Binger T, Meyer B, et al Assays for laboratory confirmation of novel human coronavirus (hCoV-EMC) infections. Euro Surveill. 2012;17(49). 10.2807/ese.17.49.20334-en 23231891

[69] Drosten C, Gunther S, Preiser W, van der Werf S, Brodt HR, Becker S, et al Identification of a novel coronavirus in patients with severe acute respiratory syndrome. N Engl J Med. 2003;348(20):1967–76. 10.1056/NEJMoa030747 12690091

[70] Biesold SE, Ritz D, Gloza-Rausch F, Wollny R, Drexler JF, Corman VM, et al Type I interferon reaction to viral infection in interferon-competent, immortalized cell lines from the African fruit bat Eidolon helvum. PLoS One. 2011;6(11):e28131 10.1371/journal.pone.0028131 22140523 PMC3227611

[71] Pettersen EF, Goddard TD, Huang CC, Couch GS, Greenblatt DM, Meng EC, et al UCSF Chimera—a visualization system for exploratory research and analysis. J Comput Chem. 2004;25(13):1605–12. 10.1002/jcc.20084 15264254

[72] Cornish-Bowden A. Nomenclature for incompletely specified bases in nucleic acid sequences: recommendations 1984. Nucleic Acids Res. 1985;13(9):3021–30. 2582368 10.1093/nar/13.9.3021 PMC341218

