# Supplementary figures and images for: The papain-like protease determines a virulence trait that varies among members of the SARS-coronavirus species
Source: PLoS Pathog. 2018 Sep 24;14(9):e1007296. doi: 10.1371/journal.ppat.1007296 (PMC6171950; doi:10.1371/journal.ppat.1007296)

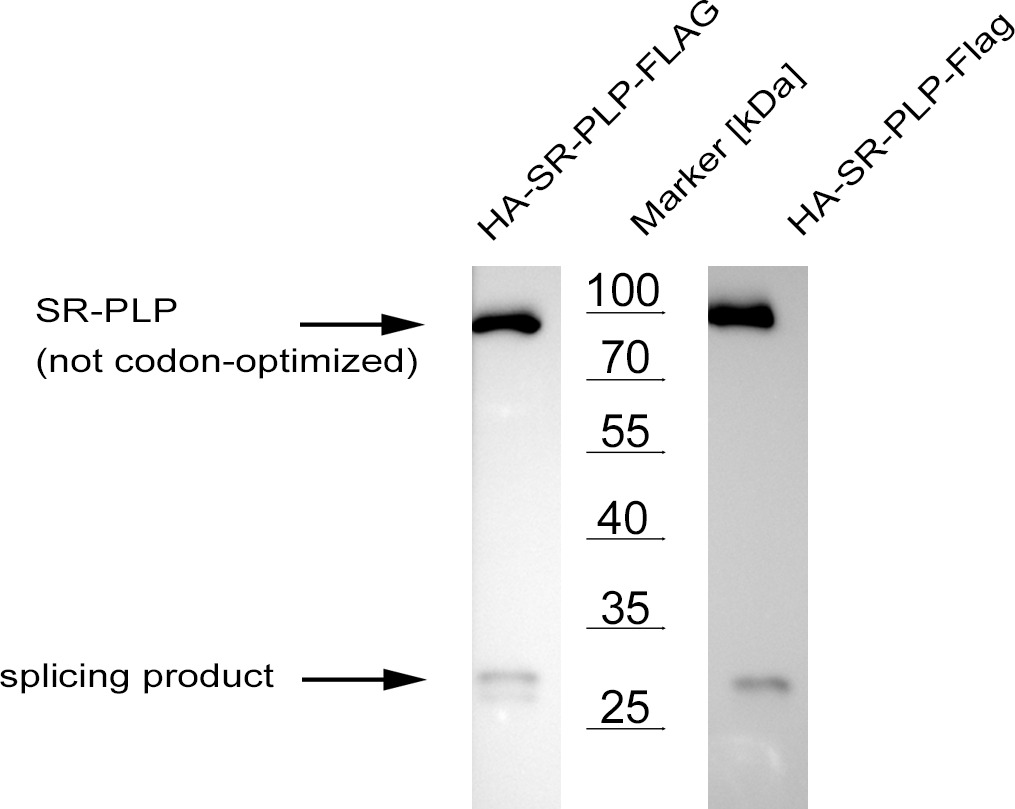

Supplement: S1 Fig — HEK-293T cells were transfected with a plasmid expressing non-optimized SR-PLP. For detection of potential splicing products the amino-terminus contained a Flag- and the carboxy-terminus contained an HA-tag. Protein expression was confirmed by Western Blot analysis using Flag- or HA-specific antibodies produced in mouse, respectively. Secondary detection was done using anti-mouse antibodies coupled with horseradish peroxidase, which were produced in goat. (TIF) [file ppat.1007296.s001.tif]

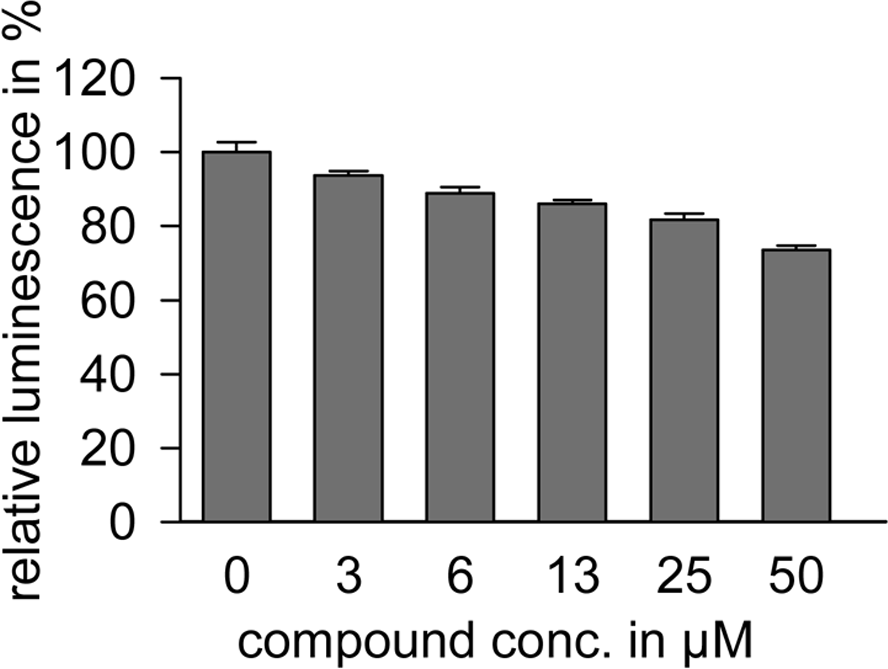

Supplement: S2 Fig — The number of viable cells after inhibitor treatment was determined by a luminescence-based cell viability assay. Therefore, Vero cells were seeded in an opaque 96-well plate and after 20 h, cells were incubated with either DMSO or a serially diluted compound 3e using DMEM as diluent. After 24 h, 100 μl of detection reagent was added and incubated for another 15 min. Emitted luminescence was recorded by a detection reader with an integration time of 1s. (TIF) [file ppat.1007296.s002.tif]
