# Supplementary material for: The papain-like protease determines a virulence trait that varies among members of the SARS-coronavirus species
Source: PLoS Pathog. 2018 Sep 24;14(9):e1007296. doi: 10.1371/journal.ppat.1007296 (PMC6171950; doi:10.1371/journal.ppat.1007296)
Supplement: S1 Table — aUnderlined nucleotides were added for cloning purposes. bFLAG tag is highlighted in bold. For: Forward primer; Rev: Reverse primer. (DOCX) [file ppat.1007296.s003.docx]

| **Application** | **Primer** | **Oligonucleotide sequence (5' to 3')^a,b^** | **Polarity** | **Designation** |
| --- | --- | --- | --- | --- |
| PLP WT | Eco_Kozak_N3plpco_small_F | CCGCCGGAATTCGCCACCATGGAAGTGAAAACCATCAAGGTGTTCGAAAACCATCAAGGTGTTC | For | SA-PLP/SR-PLP |
| PLP WT | bN3plpco_small_Flag_TGA_Not_R | ATAGTTTAGCGGCCGCTCA**CTTGTCATCGTCGTCCTTGTAGTC**TCCTGCCTTGATGTCGGTGCTGTAGCTTG | Rev | SR-PLP |
| PLP WT | sN3plpco_small_Flag_TGA_Not_R | ATAGTTTAGCGGCCGCTCA**CTTGTCATCGTCGTCCTTGTAGTC** | Rev | SA-PLP |
| PLP CA | SR-PLP_CA_5'-P_F | CATCAAGTGGGCCGACAACAACGCATACCTGAGCAGCGTGC | For | SR-PLP |
| PLP CA | SR-PLP_CA_5'-P_R | CTGGTCAGGCCACCGACTTGGGGGTACTTCC | Rev | SR-PLP |
| PLP CA | SO-PLP_CA_5'-P_F | CGTGAAGTGGACAGCCAACAACGCATACCTGAACG | For | SO-PLP |
| PLP CA | SO-PLP_CA_5'-P_R | CAGAACAGGCCGGTGTTGGGCACCTCG | Rev | SO-PLP |
| PLP CA | SARS-PLP_CA_5'-P_F | CATCAAGTGGGCCGACAACAACGCATACCTGAGCAGCGTGC | For | SA-PLP |
| PLP CA | SARS-PLP_CA_5'-P_R | CTGGTCAGGCCTCCCACTTGGGGGATCTTCC | Rev | SA-PLP |
| PLP M209R | SA-PLP-M209R-GA-F | GTGGAAGCCGTCATGTACAGAGGCACTCTGTCTTATGAC | For | SA-PLP WT/CA M209R |
| PLP M209R | SA-PLP-M209R-GA-R | GTCATAAGACAGAGTGCCTCTGTACATGACGGCTTCCAC | Rev | SA-PLP WT/CA M209R |
