# Supplementary material for: The papain-like protease determines a virulence trait that varies among members of the SARS-coronavirus species
Source: PLoS Pathog. 2018 Sep 24;14(9):e1007296. doi: 10.1371/journal.ppat.1007296 (PMC6171950; doi:10.1371/journal.ppat.1007296)
Supplement: S2 Table — SSIII one step: SuperScript III one-step RT-PCR system with Platinum Taq DNA polymerase. (DOCX) [file ppat.1007296.s004.docx]

| **Upstream-PCR** | **Forward primer** | **Reverse primer** |
| --- | --- | --- |
| 1st round (SSIII one step) | CoV-Hip2b-F4913 | CoV-Hip2b-R5370 |
| 2nd round (Platinum taq DNA polymerase) | CoV-Hip2b-F4913 | CoV-Hip2b-R5370 |
|  |  |  |
| **Downstream-PCR** |  |  |
| 1st round (SSIII one step) | CoV-Hip2b-F8807 | CoV-Hip2b-R9174 |
| 2nd round (Platinum taq DNA polymerase) | CoV-Hip2b-F8807 | CoV-Hip2b-R9119 |
|  |  |  |
| **Extension-PCR** |  |  |
| 1st round (Expand DNA polymerase) | CoV Hip2b F1 | CoV Hip2b 8897 R1 |
| 2nd round (Phusion DNA polymerase) | CoV Hip2b F2 | CoV Hip2b 8897 R1 |
