# Supplementary material for: The papain-like protease determines a virulence trait that varies among members of the SARS-coronavirus species
Source: PLoS Pathog. 2018 Sep 24;14(9):e1007296. doi: 10.1371/journal.ppat.1007296 (PMC6171950; doi:10.1371/journal.ppat.1007296)
Supplement: S3 Table — For: Forward primer; Rev: Reverse primer. The single-letter code system for degenerated bases established by the international union of pure and applied chemistry (IUPAC) was used for definition [72]. (DOCX) [file ppat.1007296.s005.docx]

| **Primer** | **Oligonucleotide sequence (5' to 3')** | **Polarity** |
| --- | --- | --- |
| CoV-Hip2b-F4913 | CACCIGCICTIAARGAIGCYTATTAT | For |
| CoV-Hip2b-R5370 | GTTGACAATRTGNGTRTARTGWCC | Rev |
| CoV-Hip2b-F8807 | TGTTTGTCCCAGTCGTGCCTTTYTGG | For |
| CoV-Hip2b-R9174 | GGGTGGTTGGTAIAAIACRTC | Rev |
| CoV-Hip2b-R9119 | GCTAARTGRCAGCAIGCRGCYTC | Rev |
| CoV Hip2b F1 | CCTCTAGGACTTATCACTCATGG | For |
| CoV Hip2b F2 | CATGGTTTGTCGCTTACAGAGG | For |
| CoV Hip2b 8897 R1 | GAGACGACACGTTTCTTGC | Rev |
| CoV Hip2b 5083 F | CTTATCTGTGACAAGTGTGG | For |
| CoV Hip2b 4971 R | GTGTAAGCCACCATAAGTGTC | Rev |
